# Harpagide alleviates neuroinflammation and cognitive dysfunction in sepsis-associated encephalopathy: Association with the MMP9/NF-κB signaling pathway

**DOI:** 10.1371/journal.pone.0362296

**Published:** 2026-11-05

**Authors:** Fen Lou, Guochun Deng, Jiejuan Chen, Yijiang Jing, Ao Wang, Mazhong Zhang, Yongping Zhang, Bin Wang

**Affiliations:** 1 The Second Clinical Medical College, Guizhou University of Traditional Chinese Medicine, Guiyang, China; 2 The Second Affiliated Hospital, Guizhou University of Traditional Chinese Medicine, Guiyang, China; 3 Guizhou Provincial People’s Hospital, Guiyang, China; 4 Guizhou Medical University, Guiyang, China; 5 Shanghai Children’s Medical Center Guizhou Hospital, Guiyang, China; Helwan University, EGYPT

## Abstract

**Background:**

Sepsis-associated encephalopathy (SAE) is a severe neurological complication of sepsis, characterized by uncontrolled neuroinflammation and cognitive decline, yet no specific therapy is currently available. Harpagide, a naturally occurring iridoid glycoside, has been reported to possess anti-inflammatory and neuroprotective activities; however, its therapeutic potential in SAE remains unexplored. This study aimed to investigate whether Harpagide exerts neuroprotective effects in SAE and to elucidate the underlying molecular mechanisms.

**Methods:**

A mouse model of SAE was established by intraperitoneal injection of lipopolysaccharide (LPS, 18 mg/kg). Cognitive performance was assessed using behavioral assays. Brain tissue morphology and neuronal survival were evaluated through histopathological examination. Glial cell activation and the production of pro-inflammatory cytokines (TNF-α, IL-1β, IL-6) were measured. Network pharmacology was employed to predict potential targets, followed by molecular docking and molecular dynamics simulations to predict binding interactions with core targets. Western blotting was performed to determine the expression levels of key proteins involved in the MMP9/NF-κB signaling pathway.

**Results:**

Treatment with Harpagide significantly improved behavioral performance, ameliorated brain pathological changes, preserved hippocampal neurons, suppressed glial activation, and reduced the levels of TNF-α, IL-1β, and IL-6 in SAE mice. Network pharmacology analysis predicted four core targets: GAPDH, CASP3, MMP9, and ICAM1. Molecular docking and molecular dynamics simulations suggested stable binding between Harpagide and MMP9. Western blot analysis further showed that Harpagide treatment was associated with reduced expression of MMP9 and phosphorylated NF-κB p65 in both hippocampal and cortical tissues.

**Conclusions:**

The present study demonstrates that Harpagide exerts neuroprotective effects in SAE, which are associated with mitigated neuroinflammation and reduced MMP9/NF-κB signaling, supporting its potential as a promising therapeutic candidate for this devastating condition.

## 1. Introduction

Sepsis-associated encephalopathy (SAE) is a frequent neurological complication of sepsis [1]. SAE manifests as inattention, cognitive dysfunction, delirium, or even coma, significantly prolonging hospitalization and increasing mortality [2–4]. Its pathogenesis is complex, involving neuroinflammation, blood-brain barrier disruption, mitochondrial dysfunction, oxidative stress, and neuronal apoptosis [5]. Among these, neuroinflammation is considered a central event [6]. Activated glial cells release large amounts of pro-inflammatory cytokines, such as TNF-α, IL-1β, and IL-6 [7,8], which further amplify the inflammatory response via signaling pathways like NF-κB [9].

Matrix metalloproteinase-9 (MMP9) plays a critical role in neuroinflammation and has been implicated in BBB disruption in various neurological conditions [10]. As a zinc-dependent endopeptidase, MMP9 degrades basal lamina components of the BBB, increases its permeability, promotes inflammatory cell infiltration, and directly participates in neuronal apoptosis [11,12]. Growing evidence indicates that targeting MMP9 effectively alleviates neuroinflammation and BBB damage in various diseases [13], and its therapeutic potential is increasingly recognized in SAE research [14,15]. MMP9 expression is transcriptionally regulated by NF-κB [16,17], while activated MMP9 can, in turn, promote NF-κB pathway activation [18], forming a positive feedback loop that exacerbates neuroinflammation [19]. Therefore, targeting the MMP9/NF-κB axis may be an effective strategy for SAE treatment.

Natural products with anti-inflammatory properties have shown neuroprotective effects in SAE [20]. Harpagide is an iridoid glycoside isolated from the traditional Chinese medicine *Scrophularia ningpoensis* Hemsl [21] (Fig 1). Recent studies have revealed that Harpagide possesses anti-inflammatory, antioxidant, and neuroprotective properties [22–24]. For instance, Harpagide was shown to alleviate Angiotensin II (Ang II)-induced neuroinflammation, neurotoxicity, and BBB disruption by inhibiting the TLR4/MyD88/NF-κB pathway [25]. Additionally, Harpagide mitigates neuronal injury induced by oxygen-glucose deprivation and reperfusion by reducing endoplasmic reticulum stress [26]. However, whether Harpagide has a therapeutic effect on SAE and whether this involves the regulation of the MMP9/NF-κB pathway remains unreported.

**Fig 1:**
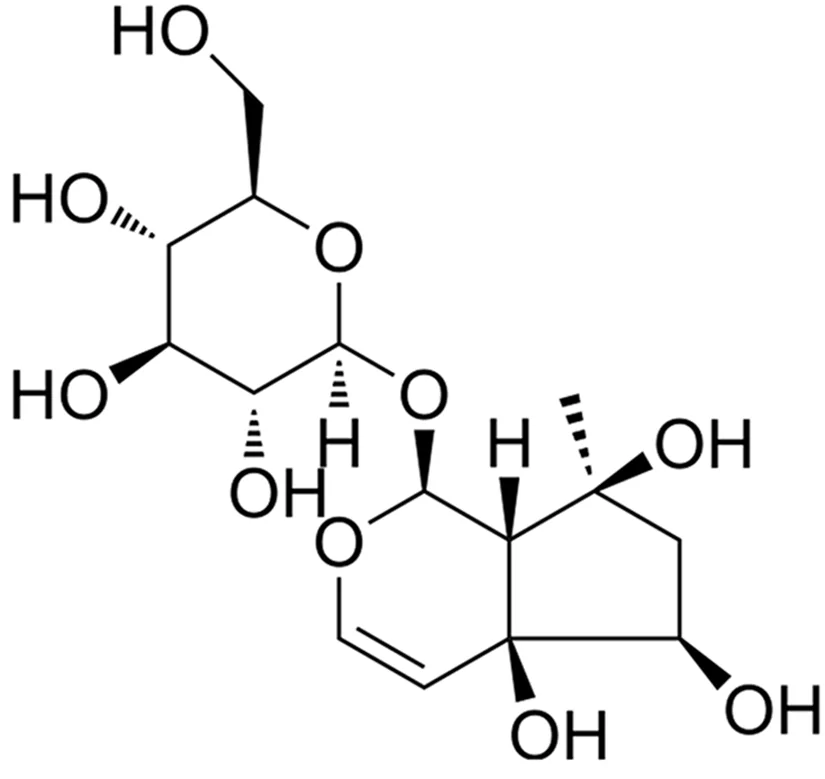
Chemical structure of Harpagide.

This study, utilizing in vivo experiments combined with network pharmacology, molecular docking, and molecular dynamics simulations, aims to investigate the therapeutic potential of Harpagide for SAE. We predicted potential targets through network pharmacology and the binding mode of Harpagide with the key target MMP9 via docking and dynamics simulations. In an LPS-induced SAE mouse model, we further validated the effects of Harpagide on behavior, histopathology, and the protein expression of key signaling molecules (MMP9 and phosphorylated NF-κB p65), providing experimental evidence for its development as a potential therapeutic agent for SAE.

## 2. Materials and methods

### 2.1 Drugs and animal management

Male C57BL/6J mice (6–8 weeks old, 20–25 g) were purchased from TQinno Biotechnology Co., Ltd (Changsha, China). All mice were housed under a 12-hour light/dark cycle at 22 ± 2°C with free access to food and water. The animal study was approved by the Experimental Animal Ethics Committee of Guizhou Medical University (Guizhou, China). Harpagide (purity ≥99%, MedChemExpress) was diluted in saline containing 2% DMSO. LPS (O55:B5, MedChemExpress) was diluted in saline.

### 2.2 Animal model and grouping

A total of 18 male C57BL/6J mice (6–8 weeks old, 20–25 g) were randomly divided into three groups (n = 6 per group): Control, LPS, and Harpagide. The LPS and Harpagide groups received an intraperitoneal (i.p.) injection of LPS (18 mg/kg) to establish the SAE model (dose based on [27], which demonstrated that this dose reliably induces cognitive deficits and neuroinflammation in mice). The Harpagide group received Harpagide (10 mg/kg) via tail vein injection immediately after LPS administration (dose based on [28]); this was administered once daily for 5 consecutive days. The Control and LPS groups received an equal volume of vehicle (saline containing 2% DMSO) via tail vein injection once daily for 5 consecutive days, matching the vehicle and injection protocol of the Harpagide-treated group. All mice were used for behavioral tests. After behavioral testing, mice were anesthetized by isoflurane inhalation (5% induction, 2–3% maintenance in 100% O_2_) and euthanized by cervical dislocation, and tissues were collected. Three mice per group were used for histopathological analysis (H&E, Nissl, and immunofluorescence), and the remaining three mice per group were used for Western blot analysis. All procedures were approved by the Experimental Animal Ethics Committee of Guizhou Medical University (Approval ID: NO.2500615).

### 2.3 Humane endpoints

This study involved a survival model in which death was a potential outcome. To minimize animal suffering, all mice were monitored every 6 hours throughout the 5-day experimental period. The following clinical signs were predefined as humane endpoints: (1) >20% weight loss from baseline; (2) inability to reach food or water; (3) moribund state (unresponsive to gentle prodding); (4) labored breathing (pronounced abdominal breathing or cyanosis). Animals meeting any criterion were immediately anesthetized with isoflurane (5% induction, 2–3% maintenance in 100% O_2_) and euthanized by cervical dislocation. No animals reached these endpoints before the scheduled end of the experiment. All researchers received training in animal handling and experimental procedures.

### 2.4 Y-maze test (Y-maze)

The Y-maze test was used to assess spontaneous alternation behavior, reflecting spatial working memory and exploratory activity. The maze consists of three white opaque arms. Each mouse was placed at the end of one arm and allowed to explore freely for 5 minutes. The sequence of arm entries was recorded and analyzed using an automated tracking system (ANY-maze, USA). Three consecutive entries into three different arms were considered a correct alternation. The alternation score was calculated as (number of correct alternations) / (total arm entries – 2). The maze was cleaned with 75% ethanol after each session to remove residual odors.

### 2.5 Open Field Test (OFT)

The OFT was used to evaluate locomotor activity, anxiety-like behavior, and exploration. The apparatus was a white opaque cubic arena (40 × 40 × 40 cm). Each mouse was placed in the center and allowed to explore for 5 minutes. The total distance traveled and the time spent in the center zone were recorded using an automated video tracking system (ANY-maze, USA). The arena was cleaned with 75% ethanol after each test.

### 2.6 Nissl staining

Whole brains were fixed in 4% paraformaldehyde for 24 h, embedded in paraffin, and coronally sectioned at 5 μm thickness. Sections were stained with Nissl staining solution (P0049, PINUOFEI). Viable neurons with Nissl bodies in the cortex were counted using ImageJ software.

### 2.7 H&E staining

Paraffin sections (6 μm thick) were deparaffinized, rehydrated through an ethanol gradient, and stained with Hematoxylin and Eosin (H&E, P0041, PINUOFEI). Pathological changes in the hippocampal tissue were observed under a light microscope (Nikon, Eclipse E100, Japan).

### 2.8 Immunofluorescence (IF) staining

After deparaffinization, rehydration, and antigen retrieval, brain sections were blocked with 10% goat serum (PN0038, PINUOFEI). Sequential multiplex fluorescence labeling was performed using Tyramide Signal Amplification (TSA) technology. Sections were incubated with rabbit anti-Iba-1 (CY7217, Abways) followed by TYR-488 (IF 488, PINUOFEI), and then with rabbit anti-GFAP (16825-1-AP, Proteintech Group) followed by TYR-555 (IF 555, PINUOFEI), with antigen retrieval between antibody rounds to elute previous antibodies. Nuclei were counterstained with DAPI. Images were captured using a laser scanning confocal microscope (3DHISTECH, PANNORAMIC, Hungary) and analyzed with ImageJ software.

### 2.9 ELISA

The concentrations of TNF-α, IL-1β, and IL-6 in hippocampal tissues were measured using commercial ELISA kits (TNF-α, E-EL-M3063; IL-1β, E-EL-M0037; IL-6, E-EL-M0044; all from Elabscience) according to the manufacturer’s instructions. Absorbance was measured at 450 nm, and sample concentrations were calculated from the standard curve.

### 2.10 Network pharmacology analysis

#### 2.10.1 Prediction of Harpagide targets.

The 3D structure of Harpagide was retrieved and downloaded from the PubChem database (https://pubchem.ncbi.nlm.nih.gov/) and submitted to the SwissTargetPrediction online platform (http://www.swisstargetprediction.ch/) for target prediction.

#### 2.10.2 Acquisition of SAE-related targets.

“Sepsis Associated Encephalopathy” was used as a keyword to search the GeneCards database (https://www.genecards.org/). Targets with a relevance score greater than 10 were selected as SAE-related targets.

#### 2.10.3 Identification of common targets.

The predicted targets of Harpagide and the SAE-related targets were imported into the Venny 2.1 online tool (https://bioinfogp.cnb.csic.es/tools/venny/) to generate a Venn diagram and identify common targets.

#### 2.10.4 Protein-protein interaction (PPI) network and core target screening.

Common targets were imported into the STRING database (https://string-db.org/), with the species set to “Homo sapiens” and a default confidence score of 0.4, to obtain PPI network data. The results were imported into Cytoscape software for topological analysis to screen for core targets based on degree centrality.

#### 2.10.5 KEGG pathway and GO enrichment analysis.

Common targets were imported into the DAVID bioinformatics resources database (https://david.ncifcrf.gov/) for KEGG pathway and Gene Ontology (GO) enrichment analysis.

### 2.11 Molecular docking and molecular dynamics simulation

#### 2.11.1 Molecular docking.

Molecular docking was performed between Harpagide and the core targets to validate the network pharmacology predictions, using binding affinity scores as the key metric. The Harpagide structure was prepared as the ligand: its 2D structure was downloaded from PubChem (https://pubchem.ncbi.nlm.nih.gov/), converted to 3D using Chem3D, and processed into the “pdbqt” format with AutoDock Tools 1.5.7. For receptor preparation, the core targets were retrieved from UniProt (https://www.uniprot.org/) for *Homo sapiens* to obtain their PDB IDs. The corresponding 3D structures were downloaded from the Protein Data Bank (PDB, https://www.rcsb.org/), dehydrated in PyMOL, and hydrogenated in AutoDock Tools 1.5.7 before export in “pdbqt” format. Docking simulations were executed using AutoDock Vina following Grid Box parameter definition. The resulting binding poses were visualized in PyMOL for the measurement and depiction of hydrogen bonds.

#### 2.11.2 Molecular dynamics simulation.

Molecular dynamics (MD) simulations were performed using GROMACS (2020.6_GPU) [29]. The Amber99SB-ILDN force field was applied for a 100 ns simulation. The system was solvated using the TIP3P water model and neutralized by adding counterions [30,31]. The distance between the solute and the box boundary was set to 1.2 nm. Energy minimization was conducted using the conjugate gradient and steepest descent methods, followed by a two-step equilibration: 200 ps NVT simulation to stabilize the temperature and 200 ps NPT simulation to equilibrate the pressure. Production MD run was performed under the NPT ensemble at 310 K and 1 bar. GROMACS tools were used to analyze the root mean square deviation (RMSD), root mean square fluctuation (RMSF), radius of gyration (Rg), solvent accessible surface area (SASA), and the number of hydrogen bonds over time. The free energy landscape was plotted to identify the most stable binding conformation. The binding free energy was calculated using the MM/PBSA method integrated within GROMACS [31].

### 2.12 Western blot

Hippocampal and cortical tissues were homogenized, centrifuged, and the supernatant was collected for protein extraction. Proteins were separated by SDS-PAGE, transferred to PVDF membranes, and blocked. Membranes were incubated overnight at 4°C with primary antibodies diluted in antibody diluent, followed by washing and incubation with HRP-conjugated secondary antibodies (Bioprimacy, PMK-014-090M). The following primary antibodies were used: anti-phospho-NF-κB p65 (11014, Signalway Antibody), anti-total NF-κB p65 (380172, Zen Bioscience), anti-MMP-9 (R24999, Zen Bioscience), and anti-GAPDH (81640–5-RR, Proteintech Group). Bands were visualized and their intensity was quantified using ImageJ software. All Western blot analyses were performed on three independent biological replicates (three individual mice per group).

### 2.13 Statistical analysis

Data were processed and analyzed using GraphPad Prism (version 10.0). Measurement data are expressed as mean ± SEM. One-way analysis of variance (ANOVA) followed by Tukey’s post hoc test was used for comparisons among three or more groups. A p-value < 0.05 was considered statistically significant.

## 3. Results

### 3.1 Harpagide ameliorates cognitive dysfunction in SAE mice

To evaluate whether Harpagide treatment improves cognitive deficits in LPS-induced septic mice, the Y-maze test was used to assess short-term spatial working memory. As shown in Fig 2C and 2D, LPS-treated mice exhibited a significant decrease in spontaneous alternation rate (p < 0.001) and total distance traveled (p < 0.001) compared to the Control group. Harpagide treatment increased both the spontaneous alternation rate (p < 0.05) and the distance traveled (p < 0.01) compared to the LPS group.

**Fig 2:**
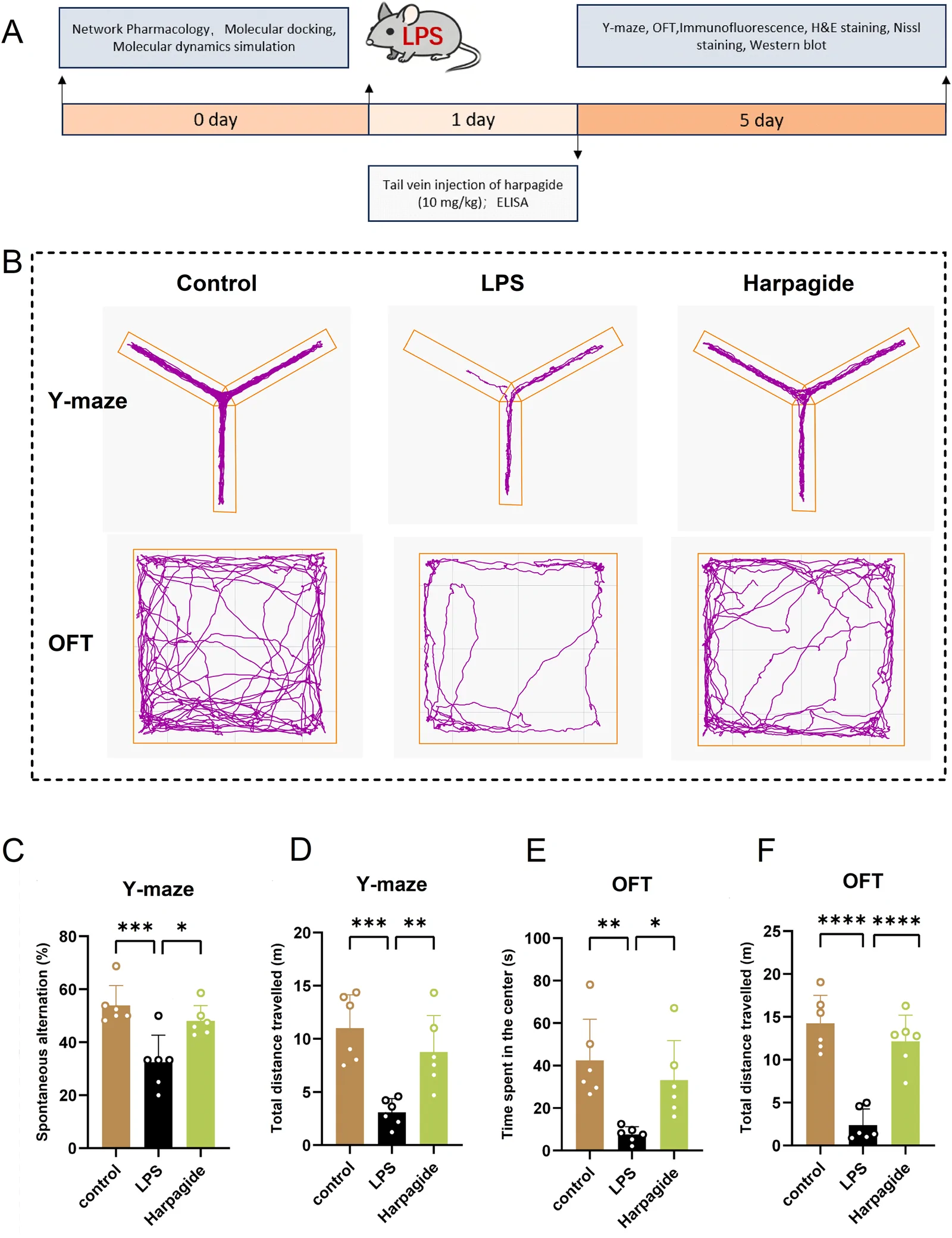
Harpagide treatment alleviates cognitive dysfunction in mice. (A) Experimental flowchart. (B) Representative movement tracks in the Open Field Test and Y-maze test. (C) Spontaneous alternation rate in the Y-maze. (D) Total distance traveled in the Y-maze. (E) Time spent in the center zone in the OFT. (F) Total distance traveled in the OFT. Tests were conducted on day 5 after treatment. n = 6 per group. Data are presented as mean ± SEM. *p < 0.05, **p < 0.01, ***p < 0.001, ****p < 0.0001.

The OFT was used to assess locomotor activity and anxiety-like behavior. As shown in Fig 2E and 2F, LPS mice spent significantly less time in the center zone (p < 0.01) and traveled a shorter total distance (p < 0.0001) compared to the Control group. Harpagide treatment significantly increased both the time spent in the center (p < 0.05) and the total distance traveled (p < 0.0001) compared to the LPS group.

These results indicate that on day 5 post-LPS modeling, mice displayed behavioral deficits characterized by reduced locomotion, lack of exploration, and increased anxiety-like behavior. Tail vein injection of Harpagide demonstrated a protective effect against these deficits.

### 3.2 Harpagide alleviates hippocampal neuronal damage and pathological injury

H&E staining of the brain, lung, liver, and kidney was performed to assess the effects of Harpagide on tissue pathology in SAE mice. As shown in Fig 3A, significant pathological changes were observed in the hippocampal CA1 region and lung tissue of the LPS group, while changes in the liver and kidney were less apparent. In the brain, the LPS group showed neuronal loss, disorganized arrangement, widened intercellular spaces (green arrows), and pyknotic nuclei (yellow arrows) in the CA1 region. In the lung, the LPS group exhibited markedly thickened alveolar septa, reduced alveolar space, structural destruction, and significant inflammatory cell infiltration and exudate around the pulmonary interstitium and airways (yellow arrows). Notably, Harpagide intervention significantly ameliorated these pathological changes.

**Fig 3:**
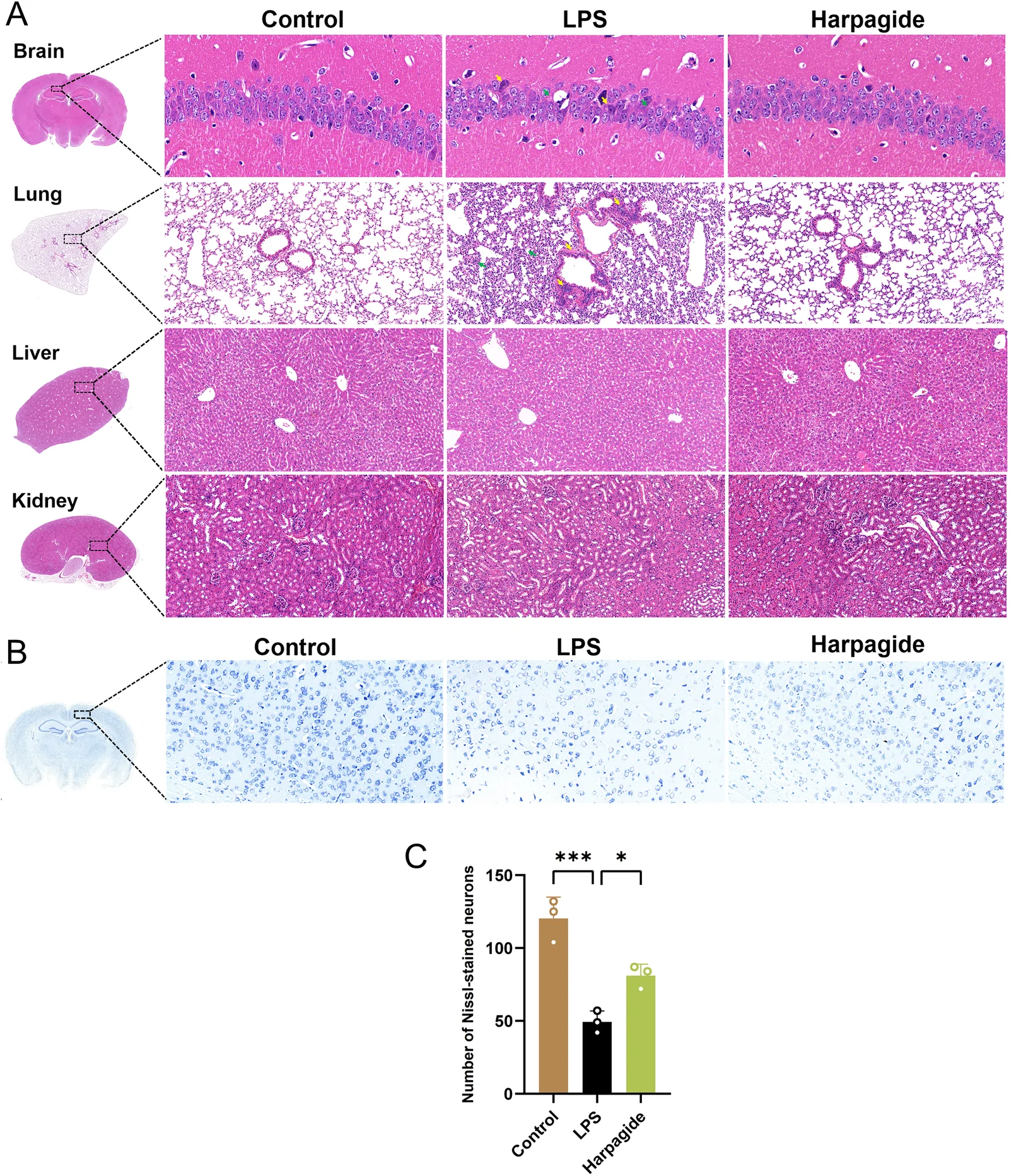
Harpagide alleviates hippocampal neuronal and pathological injury in SAE mice. (A) H&E staining of the brain (hippocampal CA1 region), lung, liver, and kidney from SAE mice after Harpagide treatment. (B) Representative images of cortical Nissl staining. (C) Quantification of Nissl-stained neurons. Scale bars: Brain 40 × , 20 μm; Lung, Liver, Kidney 20 × , 50 μm. n = 3 per group. Data are presented as mean ± SEM. *p < 0.05, **p < 0.01, ***p < 0.001, ****p < 0.0001.

Nissl staining was used to count viable neurons in the cortex. As shown in Fig 3B and 3C, the number of Nissl-stained neurons was significantly reduced in the LPS group compared to the Control group (p < 0.001), and this reduction was markedly attenuated by Harpagide treatment (p < 0.05 vs. LPS group).

These results indicate that Harpagide administration significantly improved LPS-induced neuronal damage and ameliorated pathological injury in the brain and lungs.

### 3.3 Harpagide suppresses neuroinflammation in SAE mice

The release of inflammatory cytokines promotes neuroinflammation in SAE. Therefore, ELISA was used to measure cytokine levels in the hippocampus. As shown in Fig 4A–4C, LPS induction of SAE significantly increased the levels of TNF-α (p < 0.01), IL-1β (p < 0.001), and IL-6 (p < 0.001) in hippocampal tissues compared to the Control group. Harpagide treatment significantly reduced the expression levels of these cytokines (p < 0.05 for all vs. LPS group).

**Fig 4:**
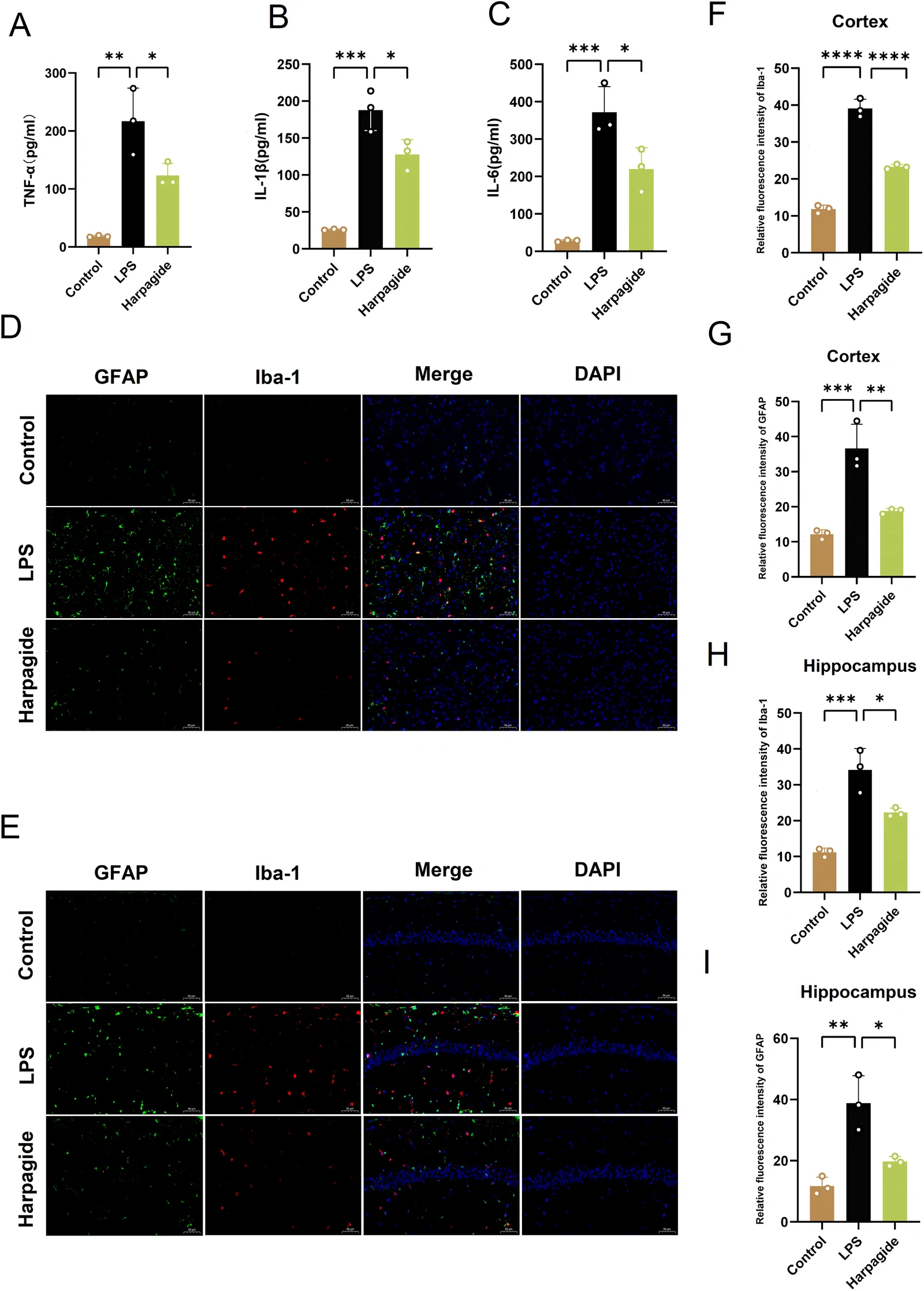
Harpagide reduces the expression levels of inflammatory cytokines and glial cell activation in LPS-induced SAE mice. (A-C) Expression levels of TNF-α, IL-1β, and IL-6 in hippocampal tissues. (D, E) Representative immunofluorescence images of Iba-1/DAPI and GFAP/DAPI staining in the cortex and hippocampal CA1 region. Scale bar = 50 μm. (F, G) Quantitative analysis of Iba-1 and GFAP fluorescence intensity in the cortex. (H, I) Quantitative analysis of Iba-1 and GFAP fluorescence intensity in the hippocampal CA1 region.n = 3 per group. Data are presented as mean ± SEM. *p < 0.05, **p < 0.01, ***p < 0.001, ****p < 0.0001.

Activation of glial cells is a central factor in SAE-associated neuroinflammation. Immunofluorescence staining for Iba-1 and GFAP was used to assess the activation of microglia and astrocytes in the hippocampus and cortex. As shown in Fig 4F and 4G, the fluorescence intensities of Iba-1 and GFAP were significantly increased in the cortex of SAE mice (p < 0.0001 and p < 0.001 vs. Control group, respectively), and these increases were significantly reduced by Harpagide treatment (p < 0.0001 and p < 0.01 vs. LPS group, respectively). Similarly, as shown in Fig 4H and 4I, the fluorescence intensities of Iba-1 and GFAP were significantly increased in the hippocampal CA1 region (p < 0.001 and p < 0.01 vs. Control group, respectively) and significantly decreased by Harpagide treatment (p < 0.05 for both vs. LPS group).

### 3.4 Network pharmacology and molecular docking reveal potential therapeutic targets

We performed an integrated network pharmacology analysis to predict the potential mechanisms of Harpagide in SAE. SwissTargetPrediction identified 78 potential targets of Harpagide. Intersection with 667 SAE-related targets from GeneCards yielded 30 common targets (Fig 5A). Protein-protein interaction (PPI) network analysis demonstrated significant interconnectivity among these common targets (Fig 5B). Subsequent topological analysis prioritized four core targets: GAPDH, CASP3, MMP9, and ICAM1 (Fig 5C). Functional enrichment analysis revealed that these targets were significantly involved in pathways such as Lipid and Atherosclerosis (KEGG, Fig 5D) and biological processes including apoptosis and cell adhesion (GO, Fig 5E). Molecular docking further predicted favorable binding affinities (binding energy < −5 kcal/mol) between Harpagide and all four core targets (Fig 5F). Based on the strong predicted binding capability with MMP9 and its central role in neuroinflammation and BBB disruption [10], MMP9 was selected for further evaluation of binding stability via molecular dynamics simulation.

**Fig 5:**
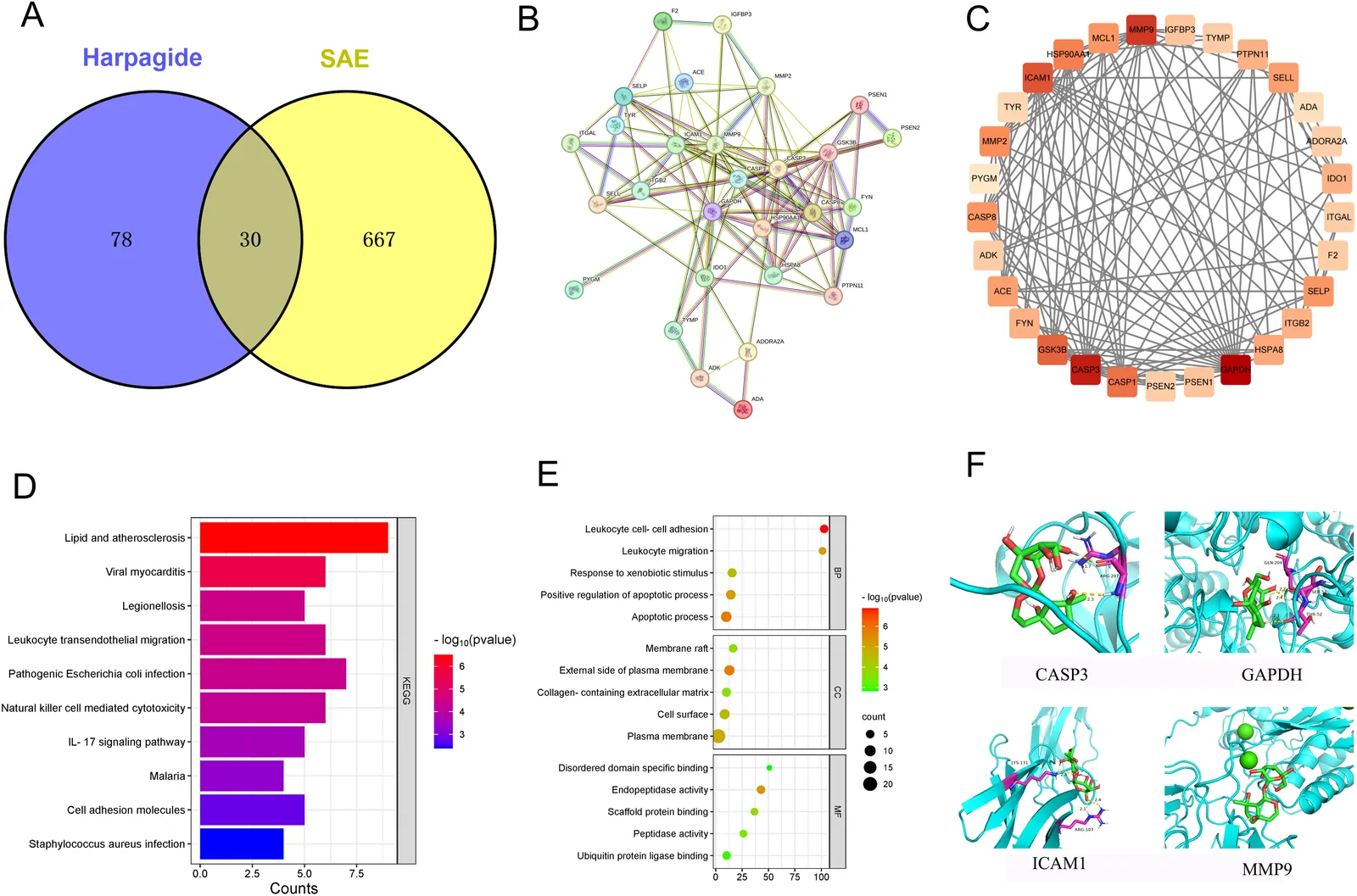
Network pharmacology and molecular docking reveal potential targets of Harpagide for SAE treatment. (A) Venn diagram of common targets between Harpagide and SAE. (B) Protein-protein interaction (PPI) network of common targets. (C) Circular layout of the PPI network highlighting core targets. (D) Top 10 KEGG pathway enrichment terms. (E) Top 5 GO enrichment terms for Biological Process (BP), Cellular Component (CC), and Molecular Function (MF). (F) Molecular docking models and binding energies of Harpagide with core targets.

### 3.5 Molecular basis revealed by molecular dynamics simulation

To evaluate the binding characteristics of Harpagide to MMP9, molecular dynamics simulation analysis was performed. The root mean square deviation (RMSD) of the complex fluctuated between 0.3–0.6 nm during the 100 ns simulation, indicating overall conformational stability (Fig 6A). Root mean square fluctuation (RMSF) suggested that the region 291–324 (particularly residue 295) exhibited higher flexibility, potentially related to ligand binding (Fig 6B). The radius of gyration (Rg) remained stable at approximately 2.36 nm, indicating consistent structural compactness (Fig 6C). Hydrogen bond analysis showed an average of approximately 2.35 stable hydrogen bonds formed between Harpagide and MMP9 (Fig 6D). The solvent accessible surface area (SASA) remained stable during the simulation, suggesting no protein unfolding (Fig 6E). The free energy landscape based on backbone RMSD and Rg showed a significant global minimum, corresponding to a stable conformational state of the system (Fig 6F and 6G).

**Fig 6:**
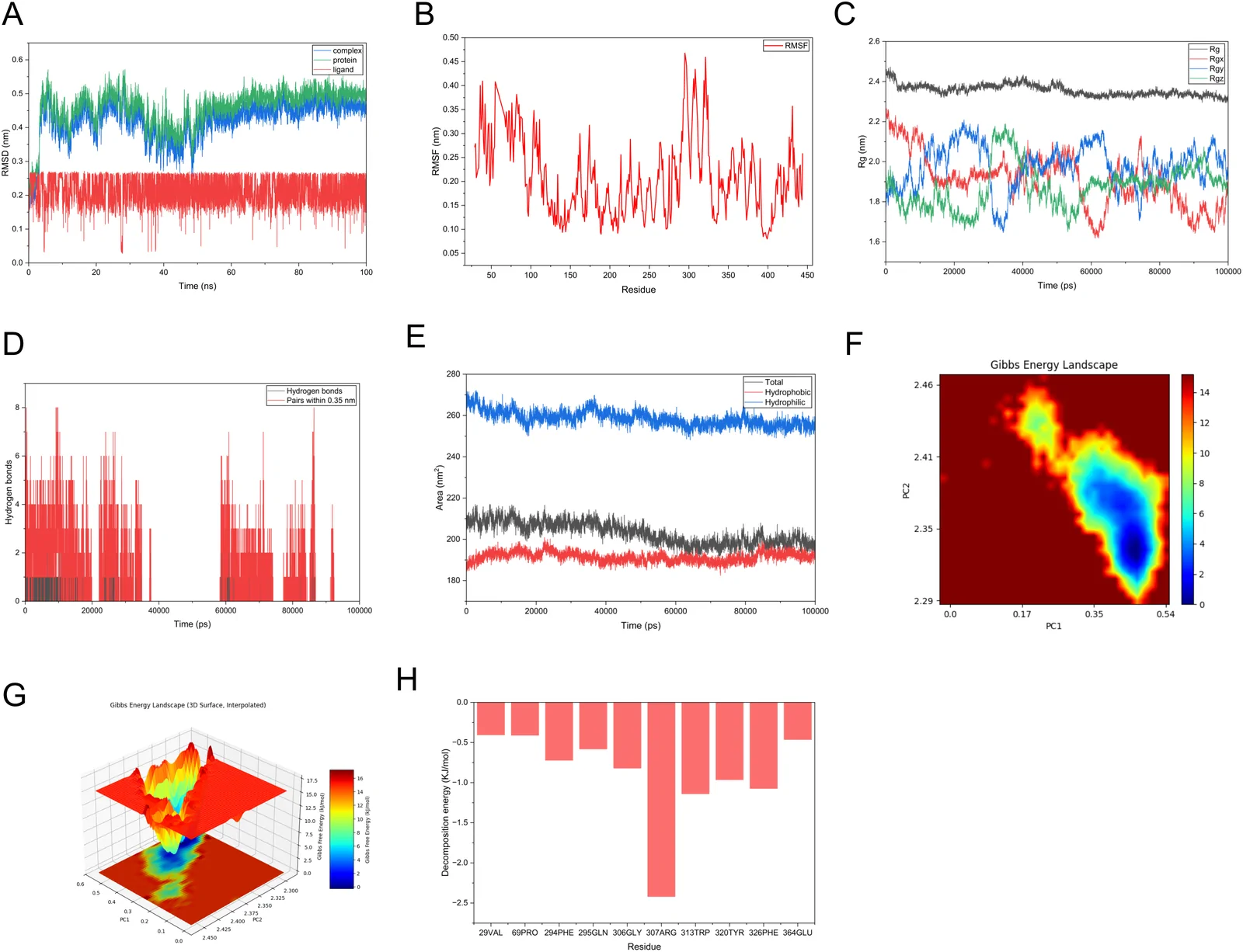
Molecular dynamics simulation reveals the binding of Harpagide to the key protein MMP9. (A) Root mean square deviation (RMSD) of the complex. (B) Root mean square fluctuation (RMSF) of the complex. (C) Radius of gyration (Rg) of the complex. (D) Number of hydrogen bonds (H-bonds) between Harpagide and MMP9 over time. (E) Solvent accessible surface area (SASA) of the complex. (F, G) Free energy landscape of the complex. (H) Energy contribution of key amino acid residues.

The binding free energy calculated using the MM/PBSA method was −35.084 kJ/mol. Analysis of the energy contribution of key residues revealed that ARG307, PHE326, TRP313, TYR320, and PHE294 contributed significantly to the binding interaction (Fig 6H), and these residues may play important roles in the catalytic process.

These results suggest that Harpagide and MMP9 can form a stable, high-affinity complex. The molecular dynamics simulations predict the molecular basis of their binding from conformational, energetic, and key residue perspectives.

### 3.6 Harpagide treatment is associated with reduced MMP9 and NF-κB protein expression in SAE mice

To further validate the target predictions from network pharmacology and molecular simulation, we examined the protein expression levels of phospho-NF-κB p65 and MMP9 in the hippocampal and cortical tissues of mice on day 5 using Western blot analysis. As shown in Fig 7B and 7C, the expression levels of phospho-NF-κB p65 (p < 0.01) and MMP9 (p < 0.001) were significantly increased in hippocampal tissues after LPS modeling compared to the Control group. However, Harpagide administration significantly reduced their levels (p < 0.01 and p < 0.001 vs. LPS group, respectively). This result was also validated in cortical tissues. As shown in Fig 7E and 7F, LPS modeling significantly increased the expression of phospho-NF-κB p65 and MMP9 in the cortex (p < 0.001 for both vs. Control group), and Harpagide administration significantly decreased their levels (p < 0.01 for both vs. LPS group). These results indicate that Harpagide treatment is associated with reduced MMP9/NF-κB signaling in the hippocampus and cortex of LPS-treated mice.

**Fig 7:**
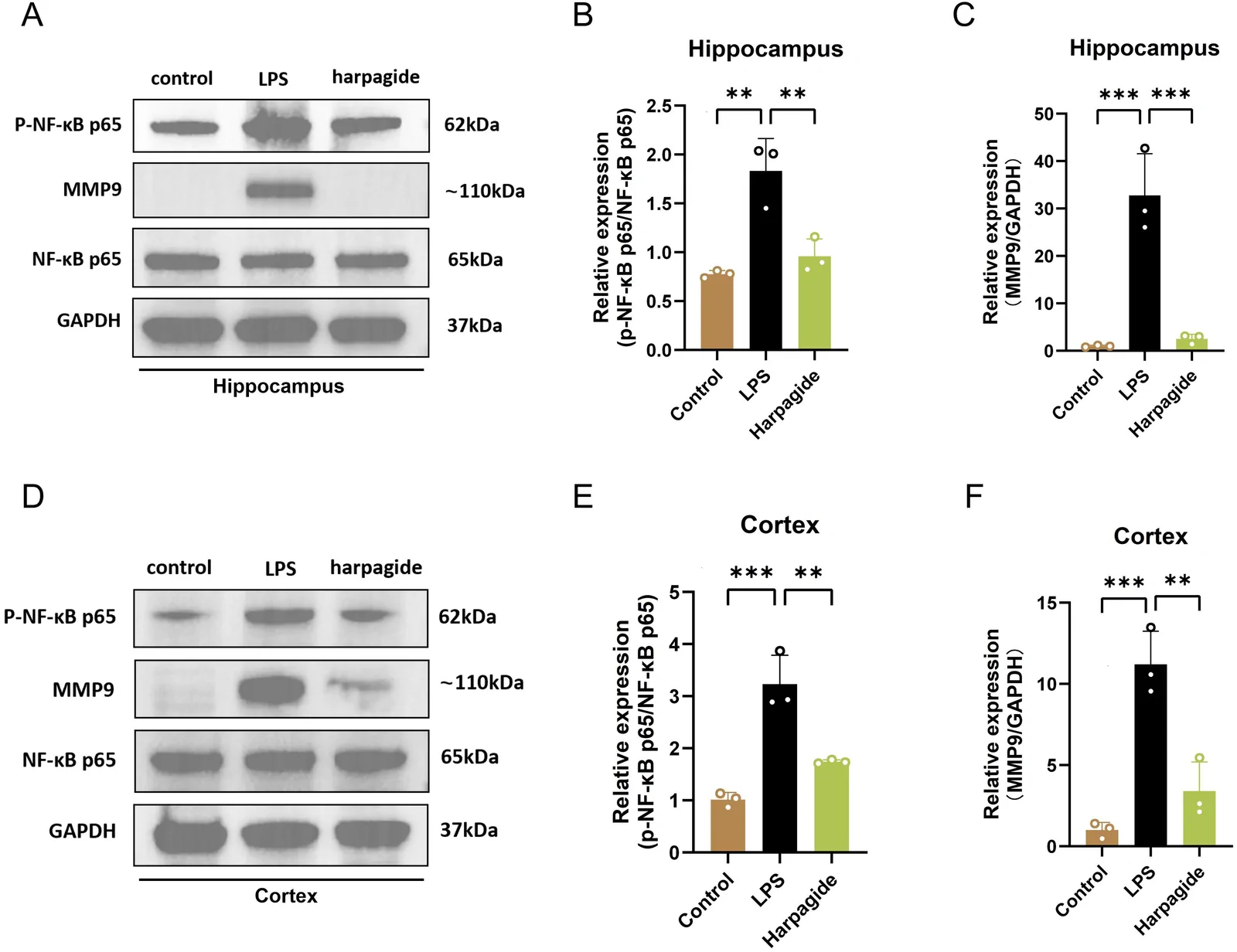
Harpagide treatment is associated with reduced MMP9 and NF-κB protein expression in SAE mice. (A, D) Representative Western blot bands of P-NF-κB p65, MMP9, total NF-κB p65, and GAPDH in mouse hippocampal and cortical tissues. (B, C) Quantitative analysis of P-NF-κB p65 and MMP9 protein bands in the hippocampus. (E, F) Quantitative analysis of P-NF-κB p65 and MMP9 protein bands in the cortex.n = 3 per group. Data are presented as mean ± SEM. *p < 0.05, **p < 0.01, ***p < 0.001, ****p < 0.0001.

## 4. Discussion

Sepsis-associated encephalopathy is a critical complication of sepsis lacking targeted therapies, with dysregulated neuroinflammation as a core pathogenic mechanism [6]. Overactivated microglia promote blood-brain barrier disruption and neuroinflammatory cascades, exacerbating brain injury [32,33]. This study demonstrates that Harpagide effectively ameliorates behavioral deficits and hippocampal neuronal damage in LPS-induced SAE mice. Its therapeutic effects are closely associated with reduced MMP9/NF-κB signaling and the mitigation of microglia-mediated neuroinflammation.

Lipopolysaccharide, a component of the Gram-negative bacterial cell wall, is a key endotoxin triggering sepsis [34,35]. Intraperitoneal injection of LPS is a well-established method for modeling SAE in animals, effectively inducing systemic inflammation that affects the central nervous system [36,37]. Its core toxic component, lipid A, is a major factor causing sepsis and multi-organ damage [38]. LPS induces systemic and central neuroinflammation through a multi-step process: it first binds to LPS-binding protein in the blood, which then presents it to the TLR4/MD-2 complex on immune cells [39,40]. TLR4 activation initiates the MyD88-dependent signaling pathway, leading to nuclear translocation of NF-κB and AP-1, which triggers the release of pro-inflammatory cytokines like TNF-α, IL-1β, and IL-6 [41,42]. Ultimately, these inflammatory signals cause sustained activation of microglia and astrocytes, creating a cerebral inflammatory microenvironment that leads to neuronal dysfunction and cognitive impairment [43,44], closely mimicking the pathological features of human SAE. In this study, LPS-modeled mice exhibited typical clinical symptoms and cognitive dysfunction, accompanied by significant neuronal damage and neuroinflammatory responses. Harpagide treatment significantly attenuated these pathological phenotypes, suggesting its neuroprotective potential.

The mechanism of Harpagide’s action is closely related to its anti-inflammatory activity [45]. The vicious cycle of neuroinflammation in SAE is primarily maintained and amplified by pro-inflammatory cytokines released from activated microglia [46]. This study showed that Harpagide treatment was associated with reduced Iba-1 fluorescence intensity, a marker of microglial activation, and decreased TNF-α, IL-1β, and IL-6 levels. Its protective effect may stem from the inhibition of microglial activation and inflammatory cytokine production. Further investigation found that this anti-inflammatory effect was associated with reduced MMP9/NF-κB signaling.

MMP9, a zinc-dependent endopeptidase, plays a key role in BBB disruption and neuroinflammation in neurological diseases [47–50]. Its expression is transcriptionally regulated by NF-κB [17], while activated MMP9 can degrade IκBα, leading to sustained NF-κB activation, forming a positive feedback loop for neuroinflammation [19]. Network pharmacology analysis predicted MMP9 as a core potential target of Harpagide. Molecular docking and dynamics simulations suggested their stable binding. In vivo experiments further showed that Harpagide treatment was associated with reduced protein expression of MMP9 and phosphorylated p65 in the hippocampus and cortex. Therefore, we speculate that Harpagide, by interacting with MMP9, may disrupt the MMP9/NF-κB feedback loop, potentially reducing NF-κB activation and pro-inflammatory cytokine release, which could contribute to the mitigation of neuroinflammation and neuronal protection. However, we acknowledge that our data demonstrate correlation rather than direct causality, and future studies using MMP9-specific inhibitors or knockout models are needed to establish direct causality.

It should be noted that the network pharmacology analysis and molecular docking were based on human SAE-related targets and Homo sapiens protein structures, whereas the experimental validation was performed in mice. This cross-species approach was adopted because human databases provide more comprehensive target information for predictive analysis. Importantly, MMP9 is highly conserved between humans and mice, and the key residues involved in the predicted Harpagide–MMP9 interaction are conserved across these species, supporting the relevance of our computational predictions to the mouse experimental model.

Previous studies have reported anti-inflammatory and neuroprotective effects of Harpagide in various models, including Ang II-induced neuroinflammation and oxygen-glucose deprivation-induced neuronal injury [25,26]. Our findings extend these observations to the context of SAE, providing the first evidence that Harpagide may be beneficial in this setting. Unlike previous in vitro studies, our work combines in vivo behavioral and molecular analyses with computational predictions to provide a more comprehensive assessment of Harpagide’s effects.

The present findings have potential translational implications. SAE currently lacks specific pharmacological treatments, and management is largely supportive [3]. The natural product Harpagide, derived from the traditional Chinese medicine *Scrophularia ningpoensis* Hemsl, represents a potential candidate for further drug development. However, the translational gap remains substantial, and future studies are needed to assess its safety, pharmacokinetics, and efficacy in preclinical models that more closely resemble clinical SAE (e.g., cecal ligation and puncture models).

Several limitations of this study should be acknowledged. First, the relatively small sample size for histological and molecular analyses (n = 3 per group) limits the statistical power and generalizability of these findings. Second, we did not directly assess BBB integrity (e.g., by Evans blue extravasation or tight junction protein expression), which limits our ability to conclude BBB protection. Third, the pharmacokinetic properties of Harpagide, including its brain penetration and potential active metabolites, were not investigated in this study. Fourth, only a single dose of Harpagide (10 mg/kg) was used; subsequent experiments with dose gradients are needed to determine the optimal therapeutic window. Fifth, although the data strongly suggest that Harpagide modulates MMP9/NF-κB signaling, establishing a direct causal relationship requires further validation using MMP9 knockout animals or specific inhibitors. Finally, the absence of a Control + Harpagide group means that we cannot exclude the possibility that repeated Harpagide administration may have effects in healthy animals. Future studies including this control group would help to further clarify the specificity of the observed effects. Addressing these limitations will provide a more solid foundation for the development of Harpagide as a potential therapeutic agent for SAE. Notably, while lower LPS doses (5–10 mg/kg i.p.) have been successfully used in some SAE models, our pilot experiments under the present experimental conditions indicated that these doses were insufficient to sustain the neuroinflammatory and behavioral responses over the required 5-day observation period. Since our experimental design aimed to evaluate the therapeutic effects of Harpagide over 5 days, the 18 mg/kg i.p. dose was selected to ensure a stable and reproducible SAE phenotype throughout the study duration.

Future research should focus on several important questions. It remains to be determined whether Harpagide directly binds to MMP9 in vivo, and whether genetic or pharmacological ablation of MMP9 would abolish the protective effects observed in this study. In addition, the optimal dosing regimen and therapeutic window of Harpagide in SAE require systematic investigation through dose-response studies. Furthermore, comprehensive pharmacokinetic profiling, including brain penetration and metabolic fate of Harpagide, is essential to better understand its in vivo behavior and to support its translational potential. Addressing these questions will be critical for advancing Harpagide toward clinical application in SAE.

## 5. Conclusion

This study provides evidence that Harpagide alleviates microglia-mediated neuroinflammation, improves behavioral deficits, and reduces pathological injury in LPS-induced SAE mice, which is associated with reduced MMP9/NF-κB signaling (Fig 8). These findings provide preclinical evidence for Harpagide as a potential therapeutic agent for SAE and also suggest a new direction for the development of neuroinflammatory inhibitors.

**Fig 8:**
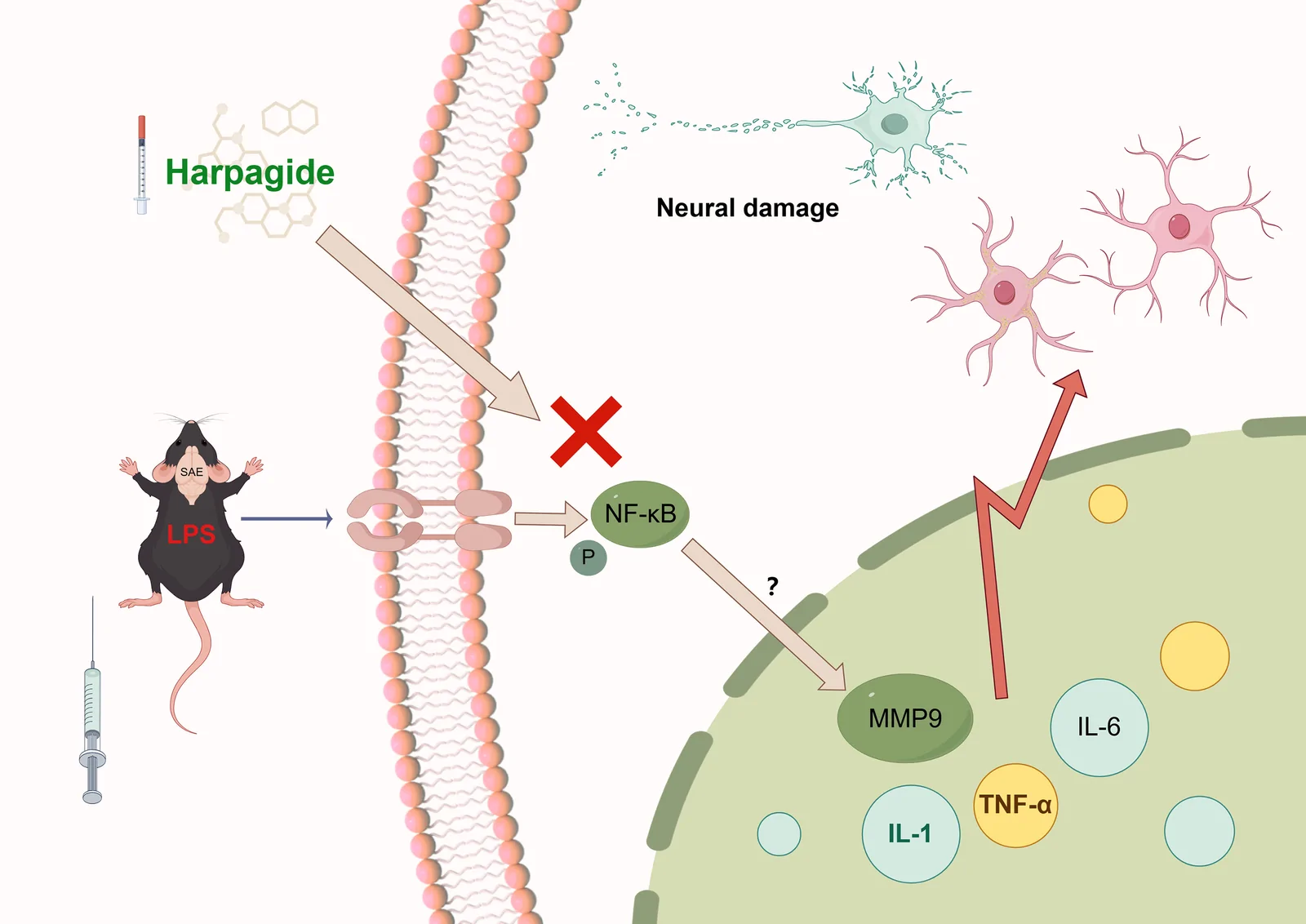
Schematic diagram illustrating the anti-inflammatory and neuroprotective effects of Harpagide associated with reduced MMP9/NF-κB signaling.

## Supporting information

S1 File Original, uncropped Western blot membrane images for MMP9, phosphorylated NF-κB p65, and total NF-κB p65 in hippocampal and cortical tissues, corresponding to the cropped blots presented in Fig 7. (PDF)

S2 File Computational data including network pharmacology (protein-protein interaction network, KEGG pathway enrichment, Gene Ontology enrichment, core target identification) and molecular docking (binding energy scores for Harpagide with CASP3, GAPDH, ICAM1, and MMP9), corresponding to Fig 5. (XLSX)

S3 File Raw experimental data for behavioral tests (Open Field Test and Y-maze), enzyme-linked immunosorbent assay (TNF-α, IL-1β, IL-6 concentrations), immunofluorescence (Iba-1 and GFAP intensities in cortex and hippocampus), and Nissl staining (viable neuron counts in cortex), corresponding to Figs 2–4. (XLSX)

## References

[1] Gofton TE, Young GB. Sepsis-associated encephalopathy. Nat Rev Neurol. 2012;8(10):557–66. doi: 10.1038/nrneurol.2012.183 22986430

[2] Gao Q, Hernandes MS. Sepsis-associated encephalopathy and blood-brain barrier dysfunction. Inflammation. 2021;44(6):2143–50. doi: 10.1007/s10753-021-01501-3 PMC11044530 34291398

[3] Mazeraud A, Righy C, Bouchereau E, Benghanem S, Bozza FA, Sharshar T. Septic-associated encephalopathy: A comprehensive review. Neurotherapeutics. 2020;17(2):392–403. doi: 10.1007/s13311-020-00862-1 PMC7283452 32378026

[4] Piva S, Bertoni M, Gitti N, Rasulo FA, Latronico N. Neurological complications of sepsis. Curr Opin Crit Care. 2023;29(2):75–84. doi: 10.1097/MCC.0000000000001022 36794932 PMC9994816

[5] Li J, Jia Q, Yang L, Wu Y, Peng Y, Du L, et al. Sepsis-associated encephalopathy: Mechanisms, diagnosis, and treatments update. Int J Biol Sci. 2025;21(7):3214–28. doi: 10.7150/ijbs.102234 40384873 PMC12080397

[6] Ren C, Yao R-Q, Zhang H, Feng Y-W, Yao Y-M. Sepsis-associated encephalopathy: A vicious cycle of immunosuppression. J Neuroinflammation. 2020;17(1):14. doi: 10.1186/s12974-020-1701-3 31924221 PMC6953314

[7] Yan X, Yang K, Xiao Q, Hou R, Pan X, Zhu X. Central role of microglia in sepsis-associated encephalopathy: From mechanism to therapy. Front Immunol. 2022;13:929316. doi: 10.3389/fimmu.2022.929316 35958583 PMC9361477

[8] Shulyatnikova T, Verkhratsky A. Astroglia in sepsis associated encephalopathy. Neurochem Res. 2020;45(1):83–99. doi: 10.1007/s11064-019-02743-2 30778837 PMC7089215

[9] Zhu D, Wang P, Chen X, Wang K, Wu Y, Zhang M, et al. Astrocyte-derived interleukin 11 modulates astrocyte-microglia crosstalk via nuclear factor-κB signaling pathway in sepsis-associated encephalopathy. Research. 2025;8:0598. doi: 10.34133/research.0598 39886603 PMC11780073

[10] Mubarak MM, Baba IA, Wani ZA, Kantroo HA, Ahmad Z. Matrix metalloproteinase-9 (MMP-9): A macromolecular mediator in CNS infections: A review. Int J Biol Macromol. 2025;311(Pt 2):143902. doi: 10.1016/j.ijbiomac.2025.143902 40328391

[11] Rempe RG, Hartz AMS, Bauer B. Matrix metalloproteinases in the brain and blood-brain barrier: Versatile breakers and makers. J Cereb Blood Flow Metab. 2016;36(9):1481–507. doi: 10.1177/0271678X16655551 27323783 PMC5012524

[12] Lu W, Wen J. The relationship among H2S, neuroinflammation and MMP-9 in BBB injury following ischemic stroke. Int Immunopharmacol. 2025;146:113902. doi: 10.1016/j.intimp.2024.113902 39724730

[13] Yoo J, Lee J, Ahn B, Han J, Lim MH. Multi-target-directed therapeutic strategies for Alzheimer’s disease: Controlling amyloid-β aggregation, metal ion homeostasis, and enzyme inhibition. Chem Sci. 2025;16(5):2105–35. doi: 10.1039/d4sc06762b 39810997 PMC11726323

[14] Xin Y, Guan T, Wang G, Xiang Y, Li M, Zhao Y, et al. Monotropein alleviates sepsis-associated encephalopathy by targeting matrix metalloproteinase-9. Neuropharmacology. 2025;279:110636. doi: 10.1016/j.neuropharm.2025.110636 40812513

[15] Zhang L, Gao Y-Z, Zhao C-J, Xia J-Y, Yang J-J, Ji M-H. Reduced inhibitory and excitatory input onto parvalbumin interneurons mediated by perineuronal net might contribute to cognitive impairments in a mouse model of sepsis-associated encephalopathy. Neuropharmacology. 2023;225:109382. doi: 10.1016/j.neuropharm.2022.109382 36543316

[16] Luqman S, Pezzuto JM. NFkappaB: A promising target for natural products in cancer chemoprevention. Phytother Res. 2010;24(7):949–63. doi: 10.1002/ptr.3171 20577970

[17] Wang S, Liu Z, Wang L, Zhang X. NF-kappaB signaling pathway, inflammation and colorectal cancer. Cell Mol Immunol. 2009;6(5):327–34. doi: 10.1038/cmi.2009.43 19887045 PMC4003215

[18] Rietz A, Spiers J. The relationship between the MMP system, adrenoceptors and phosphoprotein phosphatases. Br J Pharmacol. 2012;166(4):1225–43. doi: 10.1111/j.1476-5381.2012.01917.x 22364165 PMC3417442

[19] Fan S, Du H, Yao Y, Wang H, Zhang M, Shi X, et al. Protection against stroke-induced blood-brain barrier disruption by Guanxinning injection and its active-component combination via TLR4/NF-κB/MMP9-mediated neuroinflammation. Phytomedicine. 2025;147:157162. doi: 10.1016/j.phymed.2025.157162 40876124

[20] Yang Y-S, Liu C-Y, Pei M-Q, Sun Z-D, Lin S, He H-F. Quercetin protects against sepsis-associated encephalopathy by inhibiting microglia-neuron crosstalk via the CXCL2/CXCR2 signaling pathway. Phytomedicine. 2024;134:155987. doi: 10.1016/j.phymed.2024.155987 39216299

[21] Frezza C, et al. Harpagide: Occurrence in plants and biological activities - A review. Fitoterapia. 2020;147:104764. doi: 10.1016/j.fitote.2020.104764 33122133

[22] Kim SR, Lee KY, Koo KA, Sung SH, Lee N-G, Kim J, et al. Four new neuroprotective iridoid glycosides from Scrophularia buergeriana roots. J Nat Prod. 2002;65(11):1696–9. doi: 10.1021/np0202172 12444706

[23] Mncwangi N, Chen W, Vermaak I, Viljoen AM, Gericke N. Devil’s Claw-a review of the ethnobotany, phytochemistry and biological activity of Harpagophytum procumbens. J Ethnopharmacol. 2012;143(3):755–71. doi: 10.1016/j.jep.2012.08.013 22940241

[24] Xu C, Tang Y, Yang H, Jiang S, Peng W, Xie R. Harpagide inhibits the TNF-α-induced inflammatory response in rat articular chondrocytes by the glycolytic pathways for alleviating osteoarthritis. Int Immunopharmacol. 2024;127:111406. doi: 10.1016/j.intimp.2023.111406 38142643

[25] Lu Y, Hao R, Hu Y, Wei Y, Xie Y, Shen Y, et al. Harpagide alleviate neuronal apoptosis and blood-brain barrier leakage by inhibiting TLR4/MyD88/NF-κB signaling pathway in Angiotensin II-induced microglial activation in vitro. Chem Biol Interact. 2021;348:109653. doi: 10.1016/j.cbi.2021.109653 34516974

[26] Wang K, Lou Y, Xu H, Zhong X, Huang Z. Harpagide from Scrophularia protects rat cortical neurons from oxygen-glucose deprivation and reoxygenation-induced injury by decreasing endoplasmic reticulum stress. J Ethnopharmacol. 2020;253:112614. doi: 10.1016/j.jep.2020.112614 32007630

[27] Xu Y, Shen B, Pan X, Liu C, Wang Y, Chen X, et al. Palmatine ameliorated lipopolysaccharide-induced sepsis-associated encephalopathy mice by regulating the microbiota-gut-brain axis. Phytomedicine. 2024;124:155307. doi: 10.1016/j.phymed.2023.155307 38181529

[28] Rong Y, Liu W, Zhou Z, Gong F, Bai J, Fan J, et al. Harpagide inhibits neuronal apoptosis and promotes axonal regeneration after spinal cord injury in rats by activating the Wnt/β-catenin signaling pathway. Brain Res Bull. 2019;148:91–9. doi: 10.1016/j.brainresbull.2019.03.014 30940474

[29] Van Der Spoel D, Lindahl E, Hess B, Groenhof G, Mark AE, Berendsen HJC. GROMACS: Fast, flexible, and free. J Comput Chem. 2005;26(16):1701–18. doi: 10.1002/jcc.20291 16211538

[30] Koder Hamid M, Månsson LK, Meklesh V, Persson P, Skepö M. Molecular dynamics simulations of the adsorption of an intrinsically disordered protein: Force field and water model evaluation in comparison with experiments. Front Mol Biosci. 2022;9:958175. doi: 10.3389/fmolb.2022.958175 36387274 PMC9644065

[31] Valdés-Tresanco MS, Valdés-Tresanco ME, Valiente PA, Moreno E. gmx_MMPBSA: A new tool to perform end-state free energy calculations with GROMACS. J Chem Theory Comput. 2021;17(10):6281–91. doi: 10.1021/acs.jctc.1c00645 34586825

[32] Xin Y, Tian M, Deng S, Li J, Yang M, Gao J, et al. The key drivers of brain injury by systemic inflammatory responses after sepsis: Microglia and neuroinflammation. Mol Neurobiol. 2023;60(3):1369–90. doi: 10.1007/s12035-022-03148-z 36445634 PMC9899199

[33] Denver P, Cunningham C. Microglial activation and neuroinflammation in acute and chronic cognitive deficits in sepsis. Neuropharmacology. 2025;267:110285. doi: 10.1016/j.neuropharm.2024.110285 39746541

[34] Skirecki T, Cavaillon J-M. Inner sensors of endotoxin - implications for sepsis research and therapy. FEMS Microbiol Rev. 2019;43(3):239–56. doi: 10.1093/femsre/fuz004 30844058

[35] Peng X, Luo Z, He S, Zhang L, Li Y. Blood-brain barrier disruption by lipopolysaccharide and sepsis-associated encephalopathy. Front Cell Infect Microbiol. 2021;11:768108. doi: 10.3389/fcimb.2021.768108 34804998 PMC8599158

[36] Savi FF, de Oliveira A, de Medeiros GF, Bozza FA, Michels M, Sharshar T, et al. What animal models can tell us about long-term cognitive dysfunction following sepsis: A systematic review. Neurosci Biobehav Rev. 2021;124:386–404. doi: 10.1016/j.neubiorev.2020.12.005 33309906

[37] Qin M, Gao Y, Guo S, Lu X, Zhao Q, Ge Z, et al. Establishment and evaluation of animal models of sepsis-associated encephalopathy. World J Emerg Med. 2023;14(5):349–53. doi: 10.5847/wjem.j.1920-8642.2023.088 37908801 PMC10613796

[38] Skrzypczak-Wiercioch A, Sałat K. Lipopolysaccharide-induced model of neuroinflammation: Mechanisms of action, research application and future directions for its use. Molecules. 2022;27(17):5481. doi: 10.3390/molecules27175481 36080253 PMC9457753

[39] Lucas K, Maes M. Role of the Toll Like receptor (TLR) radical cycle in chronic inflammation: Possible treatments targeting the TLR4 pathway. Mol Neurobiol. 2013;48(1):190–204. doi: 10.1007/s12035-013-8425-7 23436141 PMC7091222

[40] Gurram PC, Manandhar S, Satarker S, Mudgal J, Arora D, Nampoothiri M. Dopaminergic signaling as a plausible modulator of astrocytic toll-like receptor 4: A crosstalk between neuroinflammation and cognition. CNS Neurol Disord Drug Targets. 2023;22(4):539–57. doi: 10.2174/1871527321666220413090541 35422229

[41] Singh S, Sahu K, Singh C, Singh A. Lipopolysaccharide induced altered signaling pathways in various neurological disorders. Naunyn Schmiedebergs Arch Pharmacol. 2022;395(3):285–94. doi: 10.1007/s00210-021-02198-9 34989812

[42] Batista CRA, Gomes GF, Candelario-Jalil E, Fiebich BL, de Oliveira ACP. Lipopolysaccharide-induced neuroinflammation as a bridge to understand neurodegeneration. Int J Mol Sci. 2019;20(9):2293. doi: 10.3390/ijms20092293 31075861 PMC6539529

[43] Millán Solano MV, Salinas Lara C, Sánchez-Garibay C, Soto-Rojas LO, Escobedo-Ávila I, Tena-Suck ML, et al. Effect of systemic inflammation in the CNS: A silent history of neuronal damage. Int J Mol Sci. 2023;24(15):11902. doi: 10.3390/ijms241511902 37569277 PMC10419139

[44] Kalyan M, et al. Role of endogenous lipopolysaccharides in neurological disorders. Cells. 2022;11(24):4038. doi: 10.3390/cells11244038 PMC9777235 36552802

[45] Menghini L, Recinella L, Leone S, Chiavaroli A, Cicala C, Brunetti L, et al. Devil’s claw (Harpagophytum procumbens) and chronic inflammatory diseases: A concise overview on preclinical and clinical data. Phytother Res. 2019;33(9):2152–62. doi: 10.1002/ptr.6395 31273865

[46] Xiao D, Wang X, Liang W, Yang Y, Du Y, Liu C, et al. Convergence of sepsis-associated encephalopathy pathogenesis onto microglia. J Transl Med. 2025;23(1):622. doi: 10.1186/s12967-025-06635-8 40462103 PMC12131422

[47] Hollidge BS, Cohen CA, Akuoku Frimpong J, Badger CV, Dye JM, Schmaljohn CS. Toll-like receptor 4 mediates blood-brain barrier permeability and disease in C3H mice during Venezuelan equine encephalitis virus infection: Venezuelan equine encephalitis virus and toll-like receptor 4. Virulence. 2021;12(1):430–43. doi: 10.1080/21505594.2020.1870834 PMC7849679 33487119

[48] Zhang Y, Shu X, Zhang Y, Song C, Wu Y, Cui K, et al. Astrocyte-derived MMP-9 is a key mediator of pseudorabies virus penetration of the blood-brain barrier and tight junction disruption. Vet Res. 2025;56(1):72. doi: 10.1186/s13567-025-01486-z 40176142 PMC11963458

[49] Ji RR, Xu ZZ, Wang X, Lo EH. Matrix metalloprotease regulation of neuropathic pain. Trends Pharmacol Sci. 2009;30(7):336–40. doi: 10.1016/j.tips.2009.04.002 PMC2706286 19523695

[50] Burmeister M, Frauenstein A, Kahms M, Arends L, Gerwien H, Deshpande T, et al. Secretomics reveals gelatinase substrates at the blood-brain barrier that are implicated in astroglial barrier function. Sci Adv. 2023;9(29):eadg0686. doi: 10.1126/sciadv.adg0686 37467333 PMC10355830

