# Supplementary material for: Harpagide alleviates neuroinflammation and cognitive dysfunction in sepsis-associated encephalopathy: Association with the MMP9/NF-κB signaling pathway
Source: PLoS One. 2026 Nov 5;21(11):e0362296. doi: 10.1371/journal.pone.0362296 (PMC13739116; doi:10.1371/journal.pone.0362296)

Full unedited gel/blot for Figure.7A

P-NF-κB p65：62kDa

Repeat 1

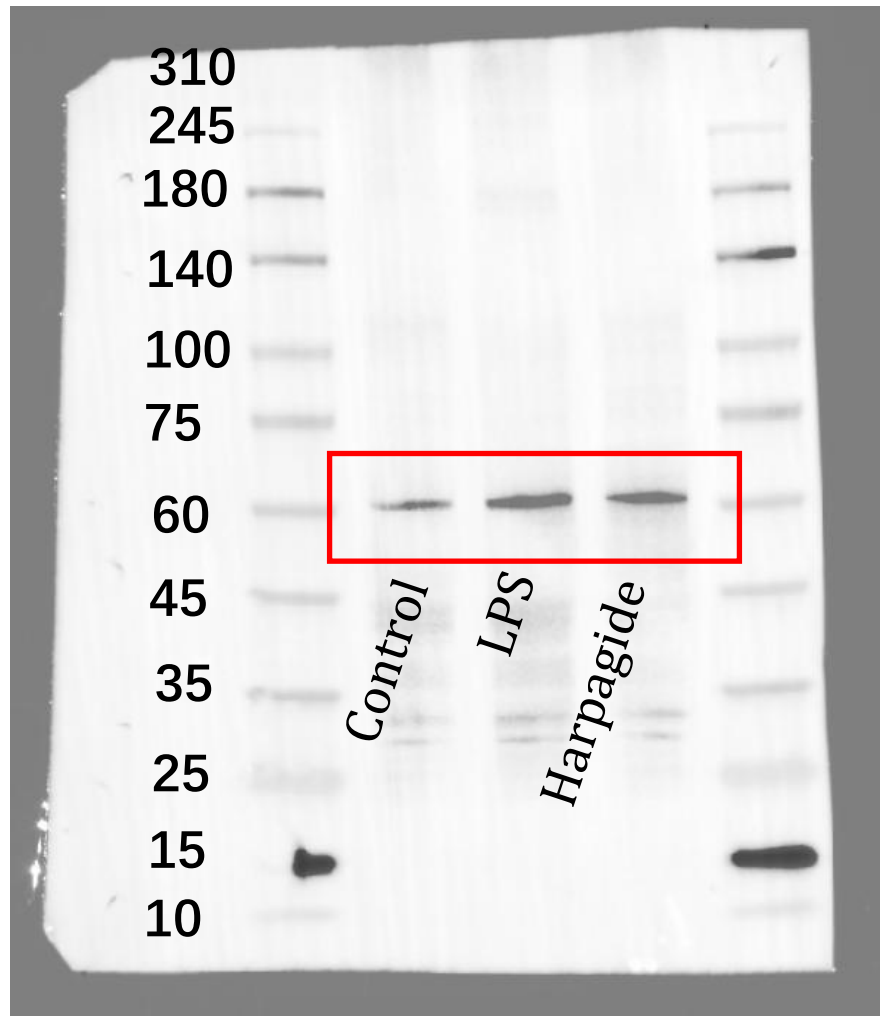

Repeat 2

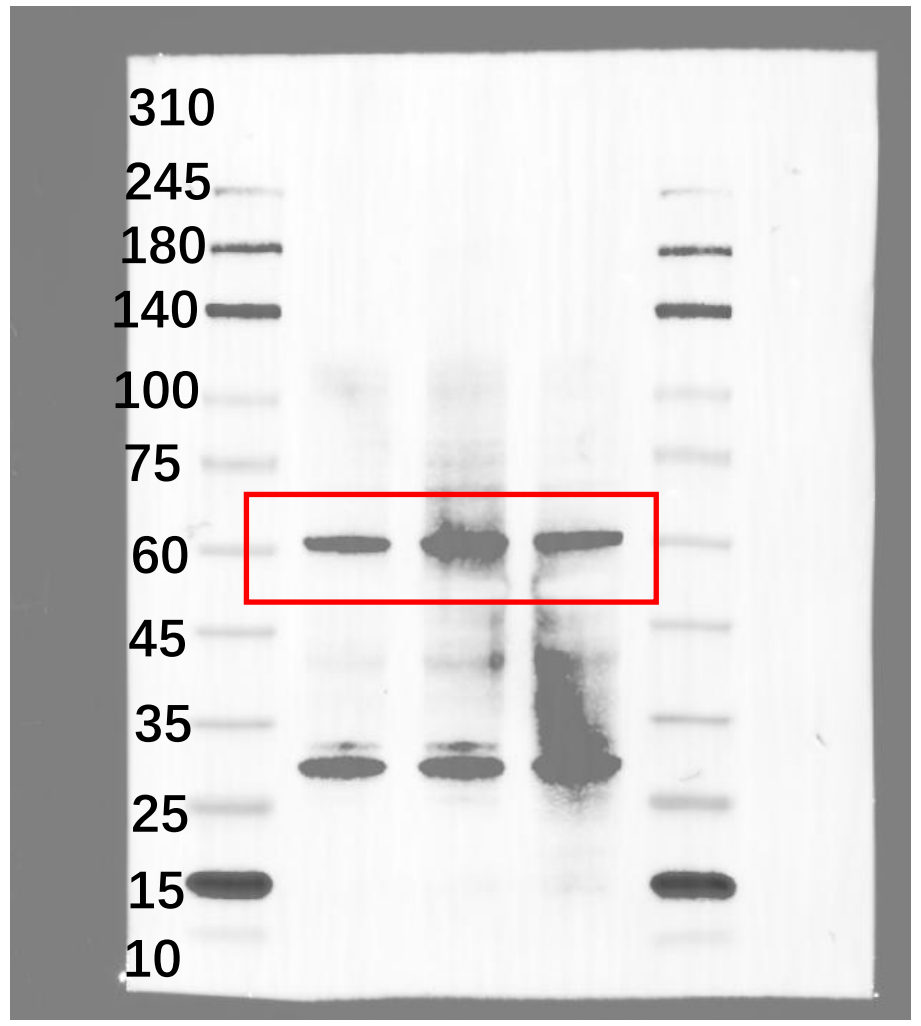

Repeat 3

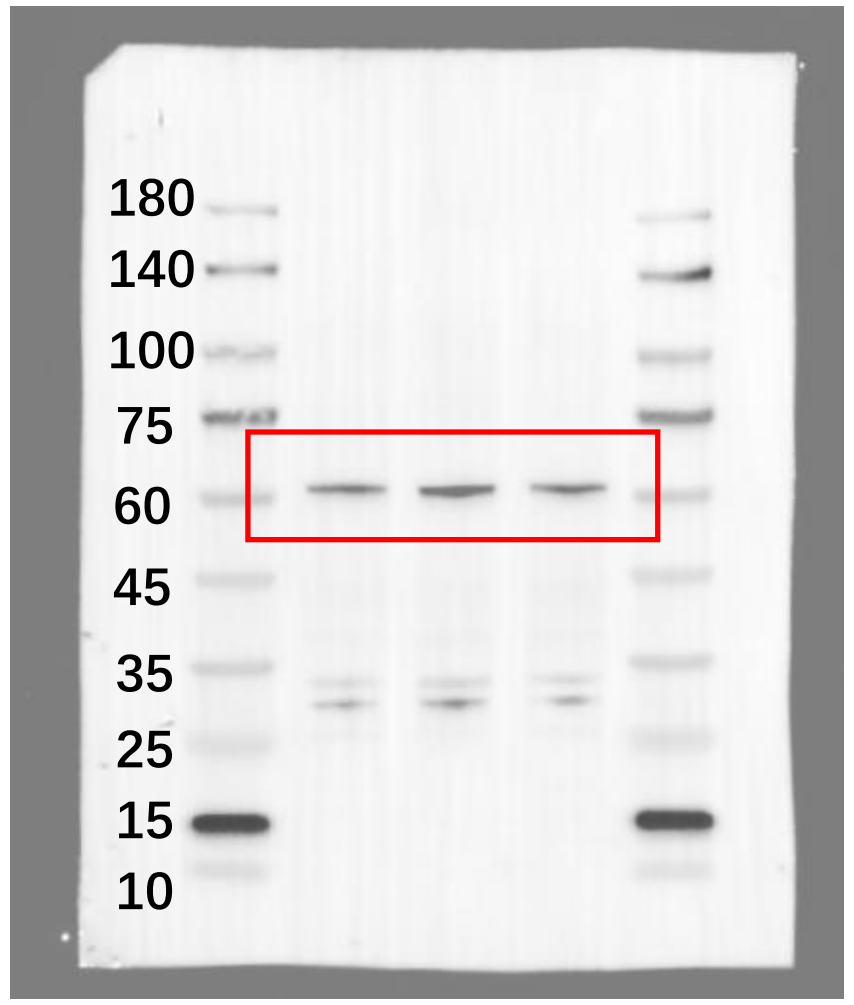

GAPDH：37kDa

Repeat 1

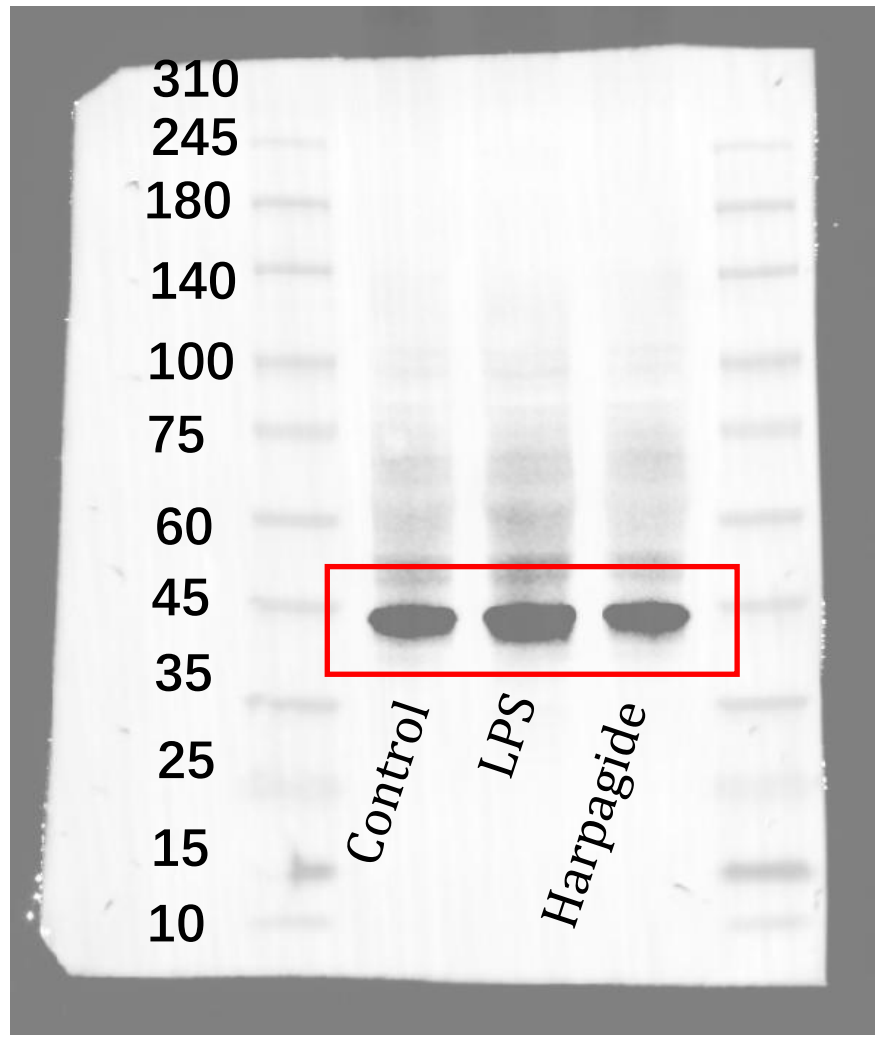

Repeat 2

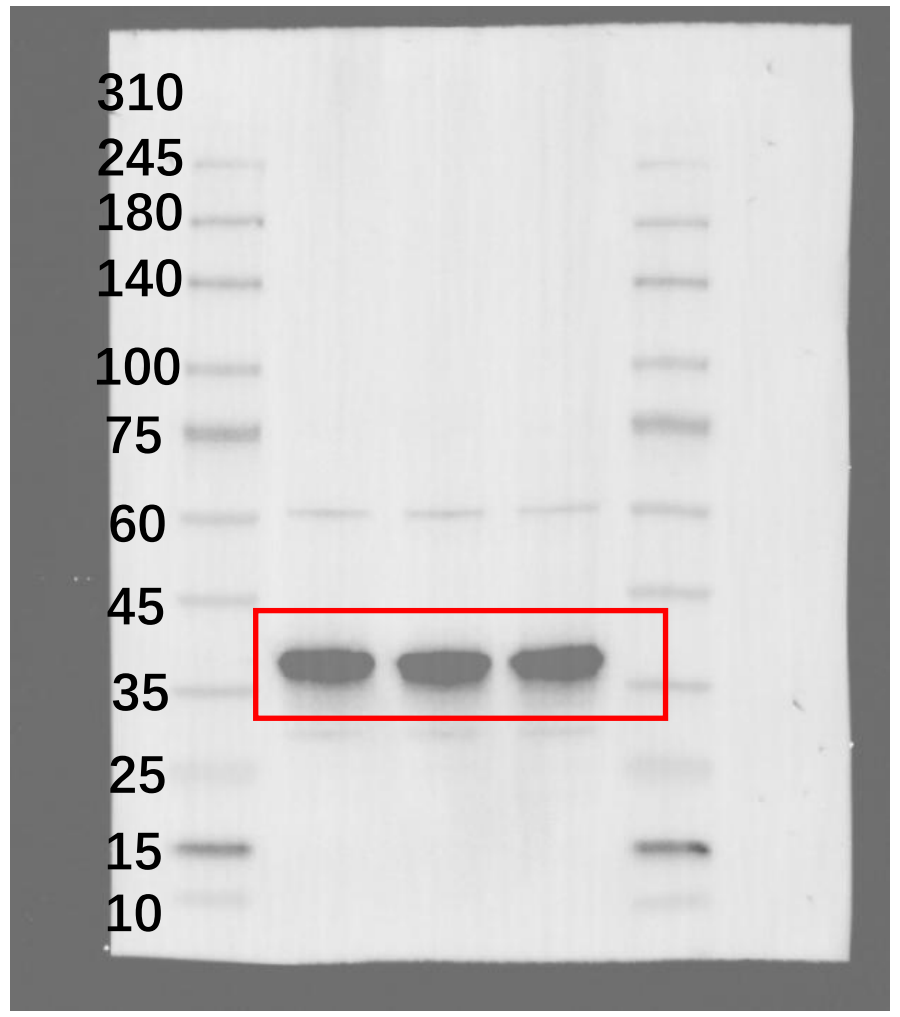

Repeat 3

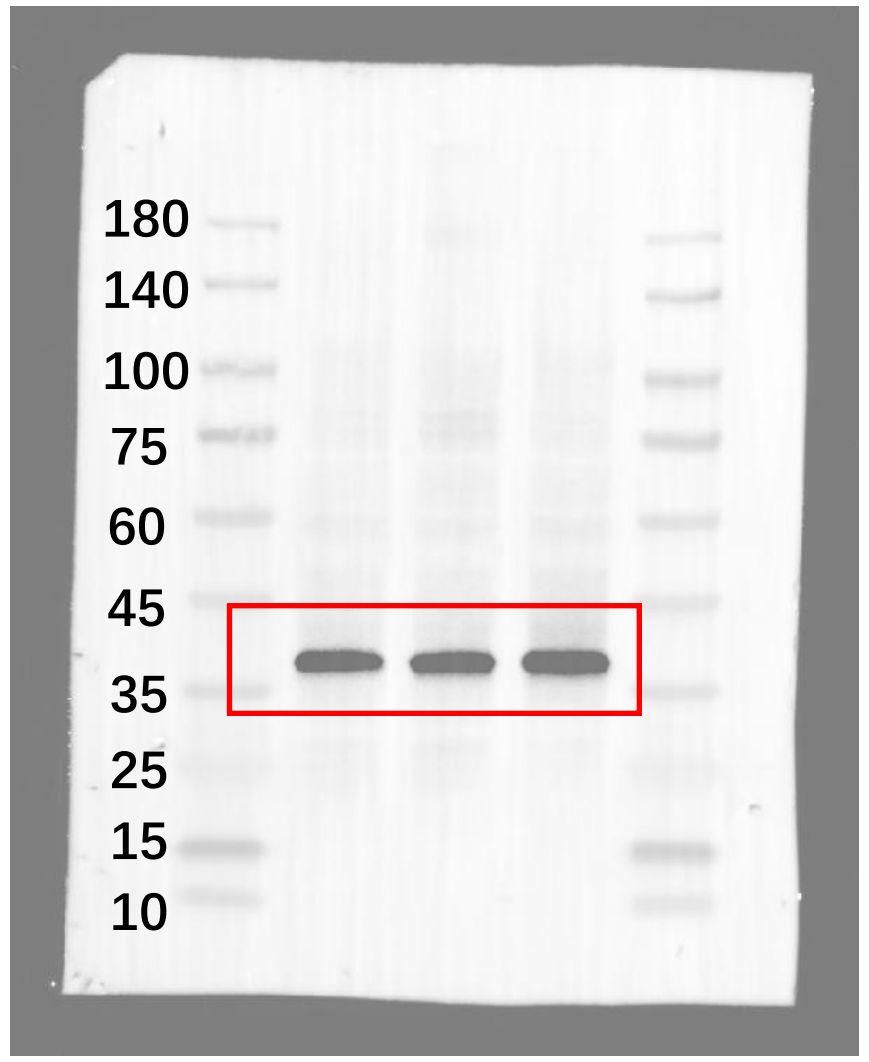

Full unedited gel/blot for Figure.7A

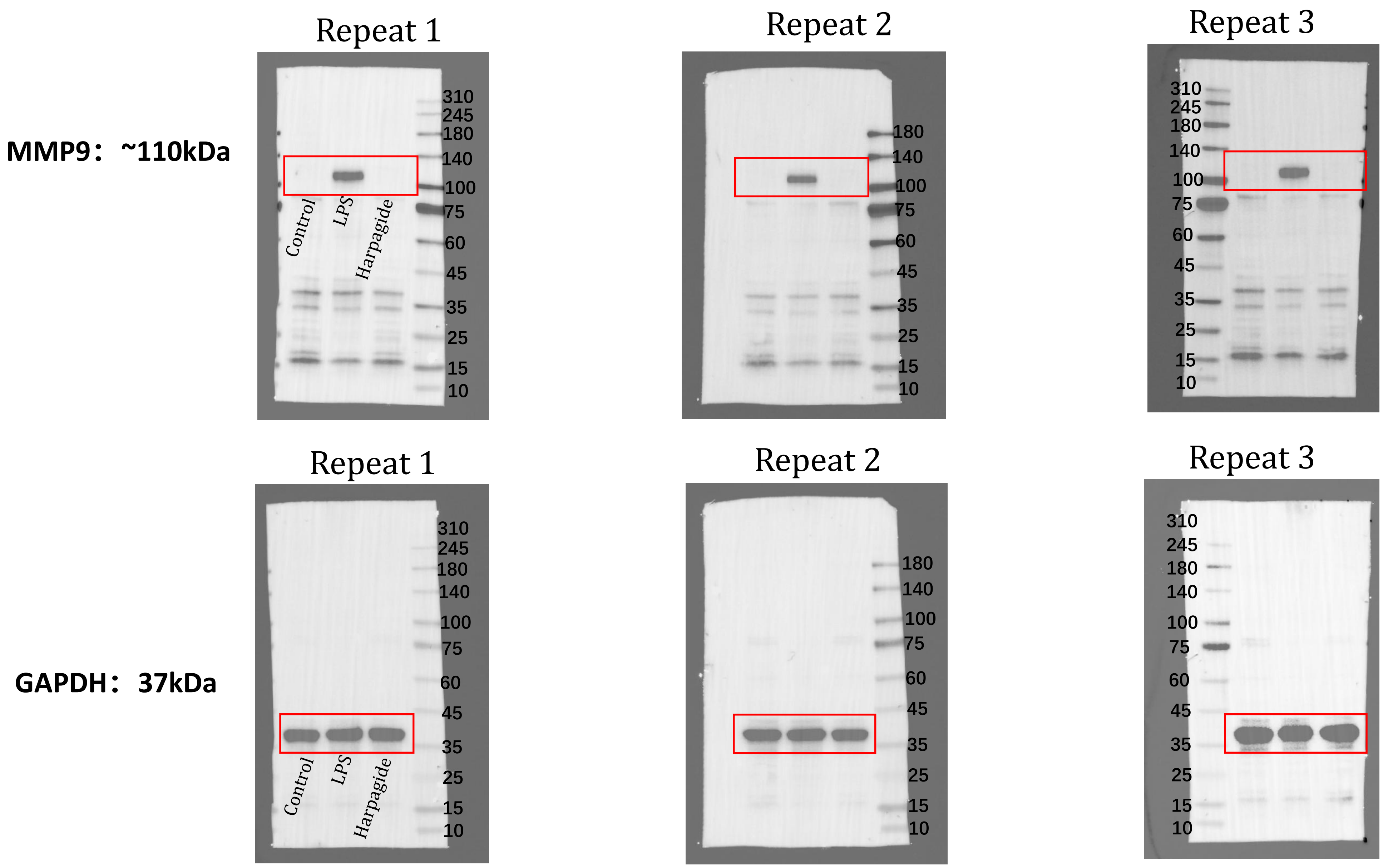

Full unedited gel/blot for Figure.7A

NF-κB p65：65kDa

Repeat 1

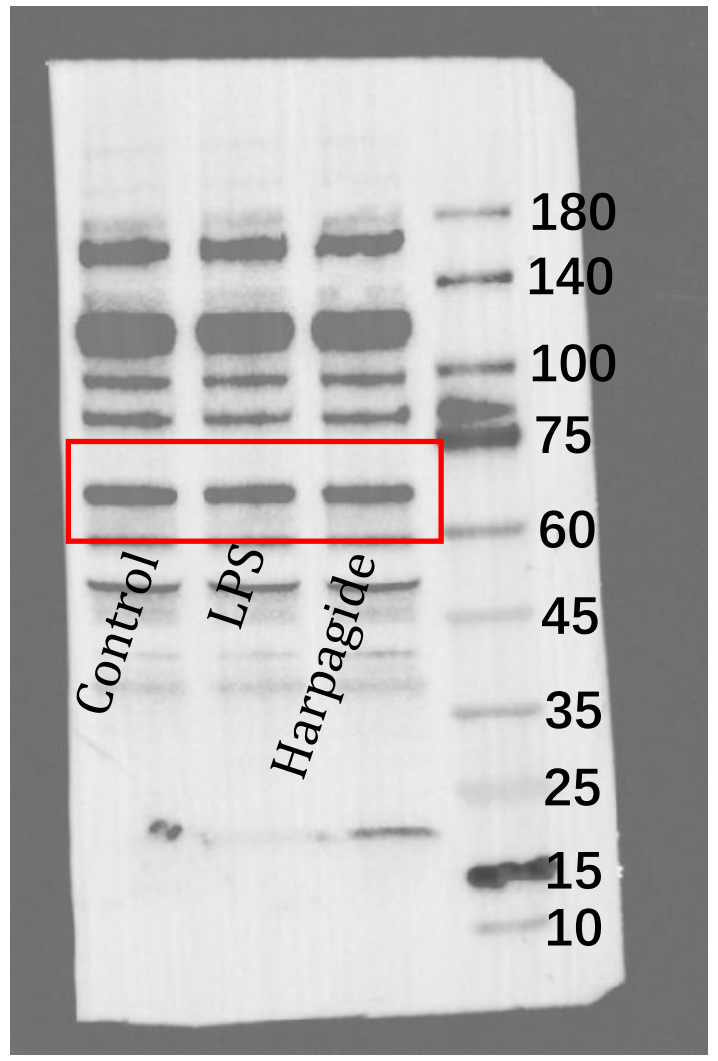

Repeat 2

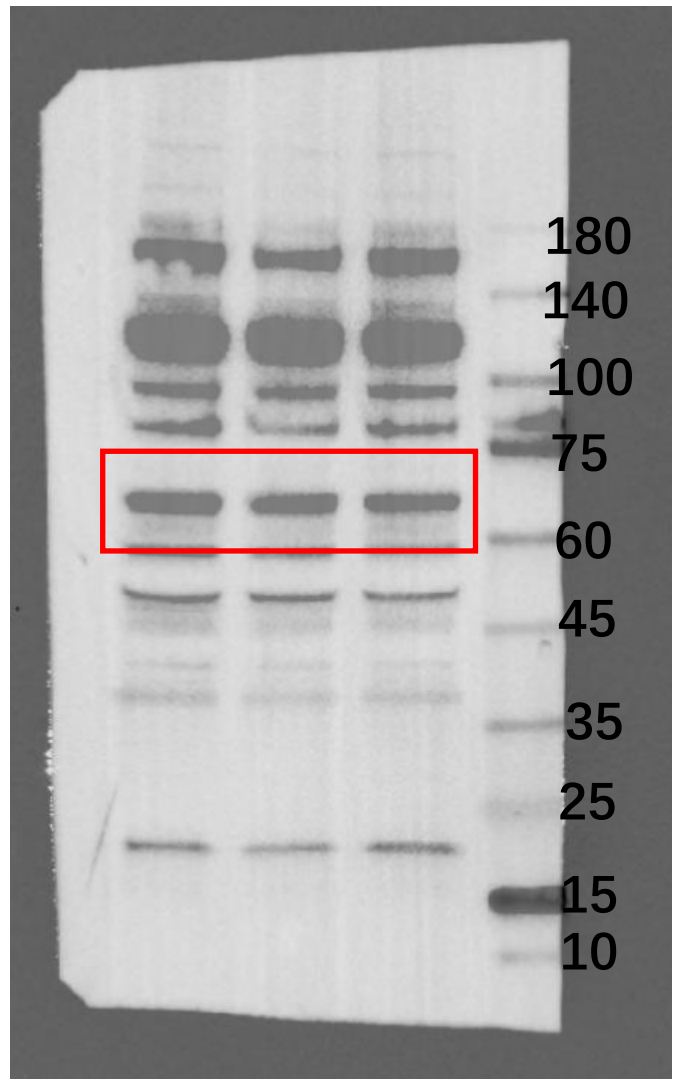

Repeat 3

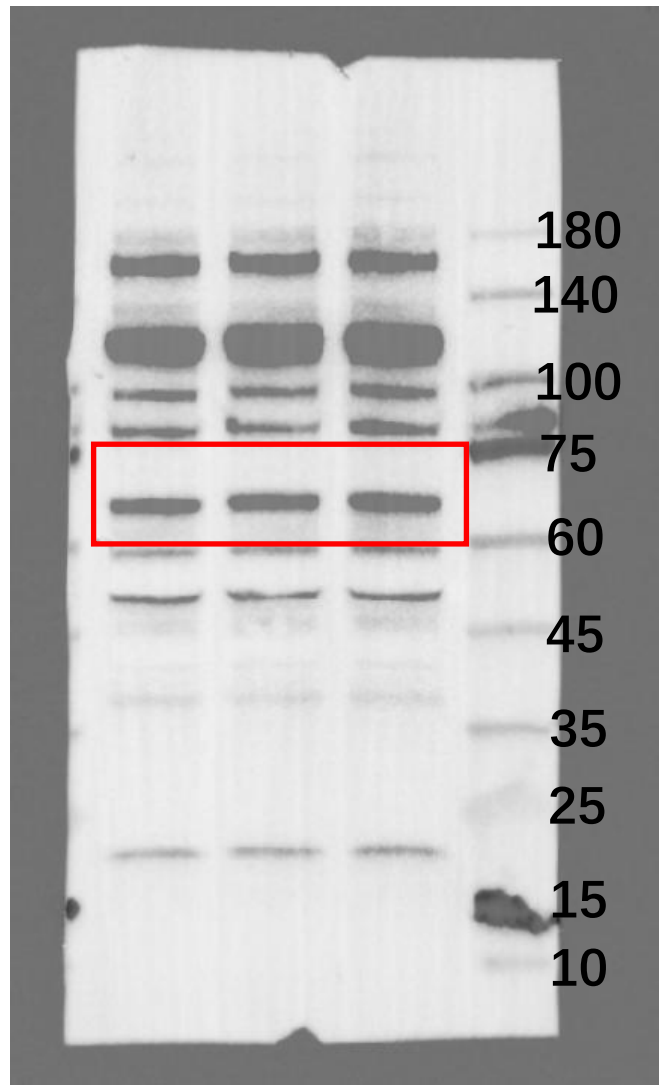

GAPDH：37kDa

Repeat 1

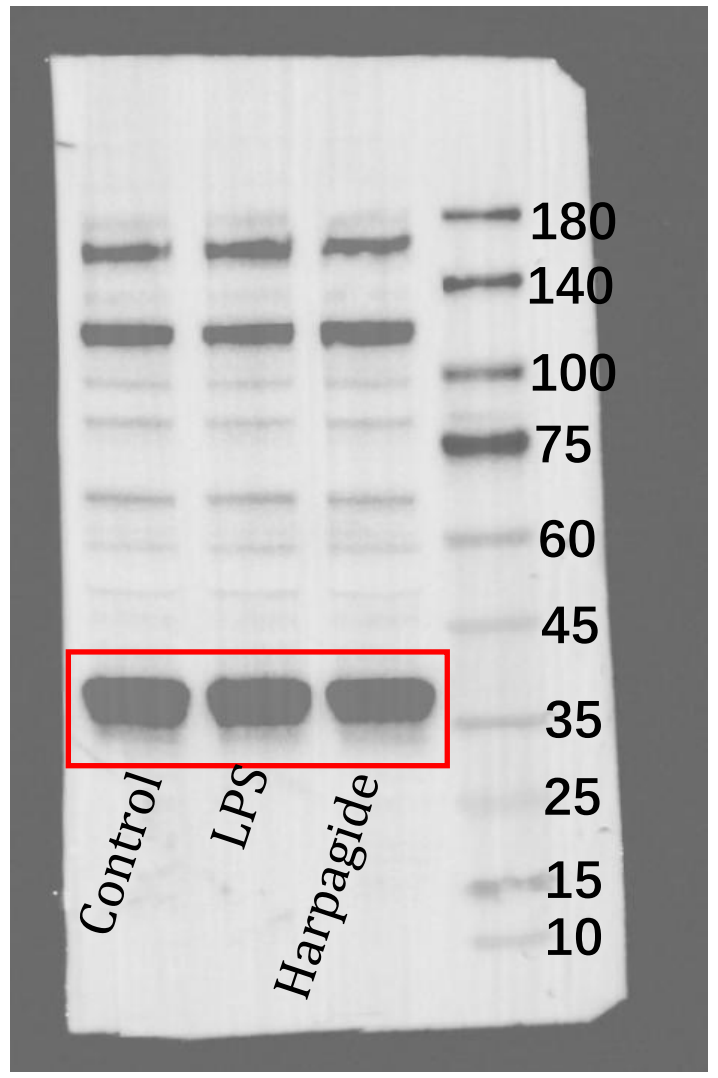

Repeat 2

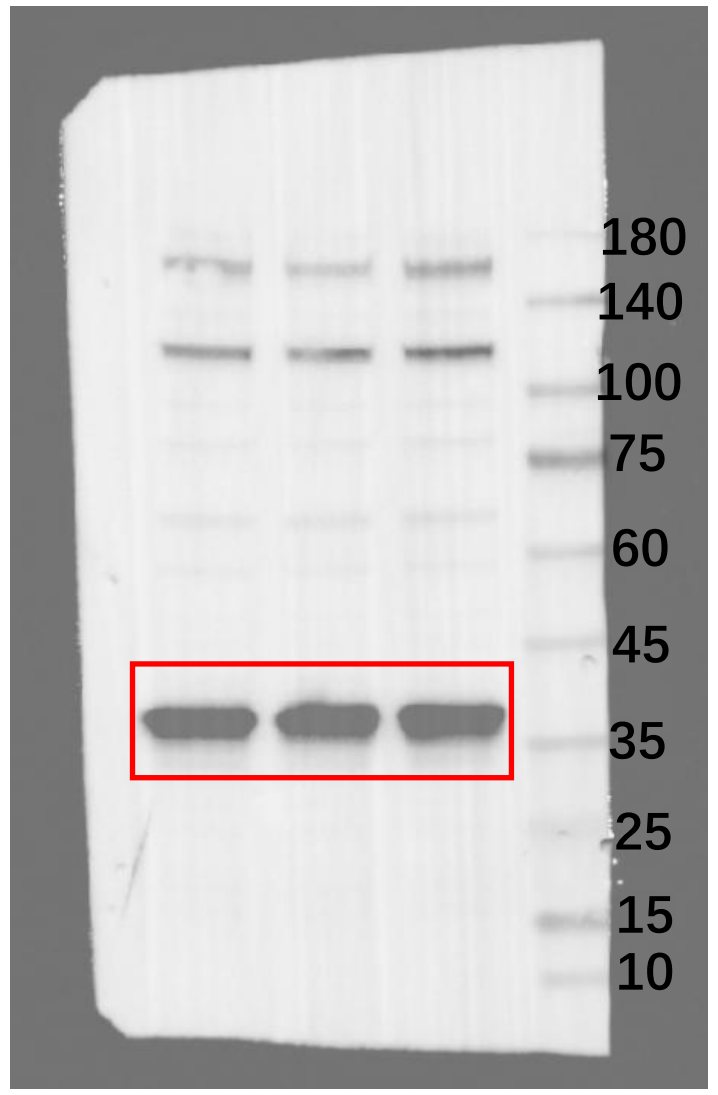

Repeat 3

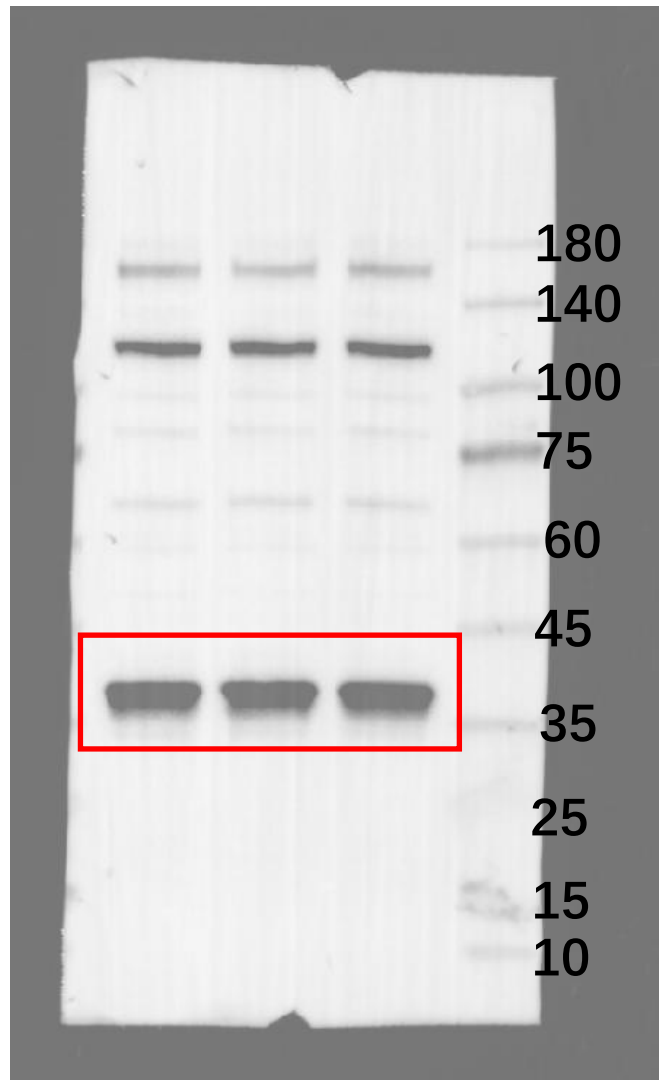

Full unedited gel/blot for Figure.7B

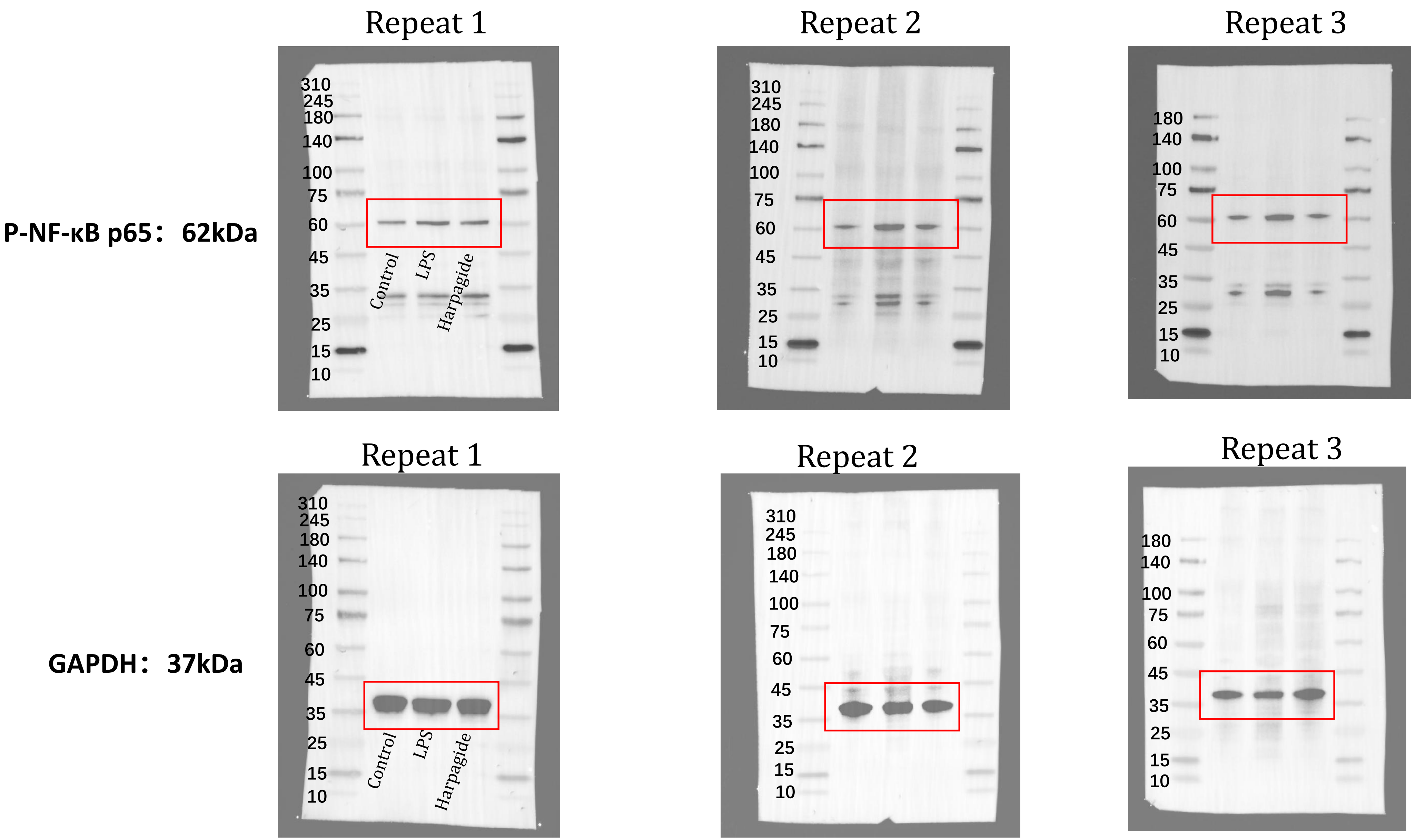

Full unedited gel/blot for Figure. 7B

MMP9： ~110kDa

Repeat 1

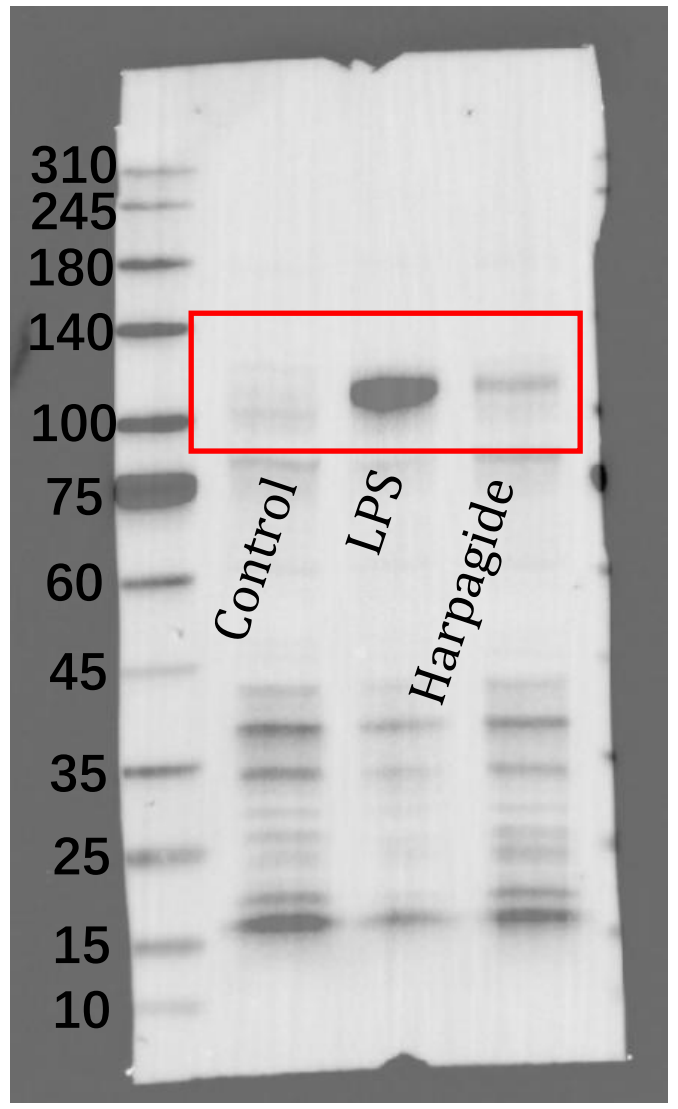

Repeat 2

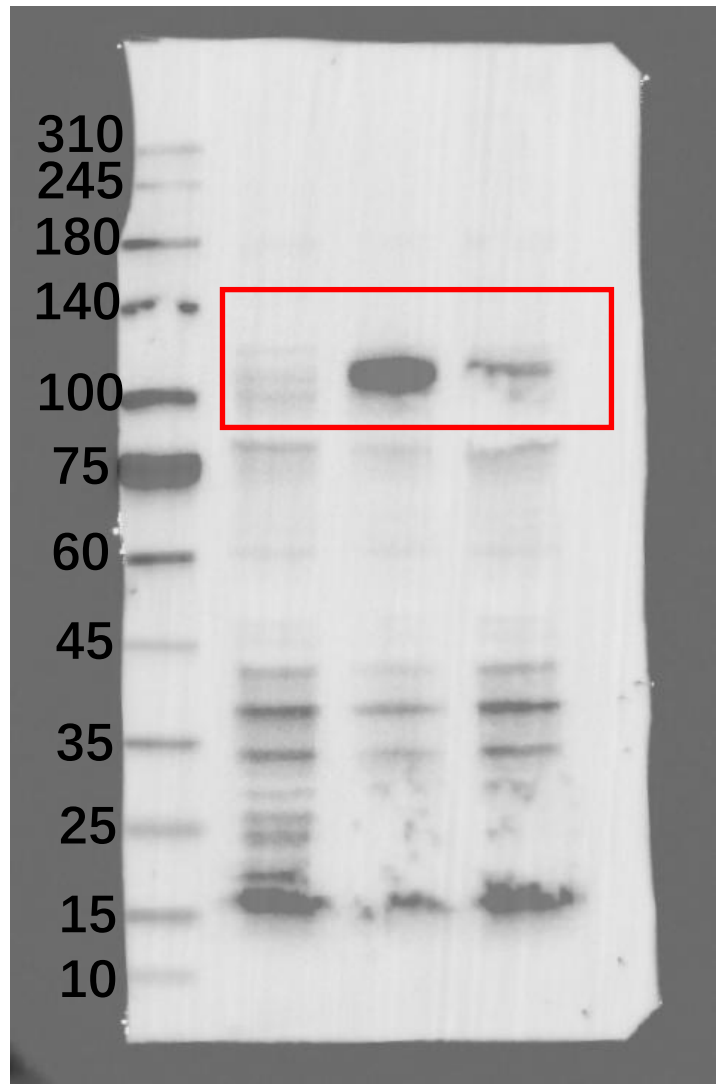

Repeat 3

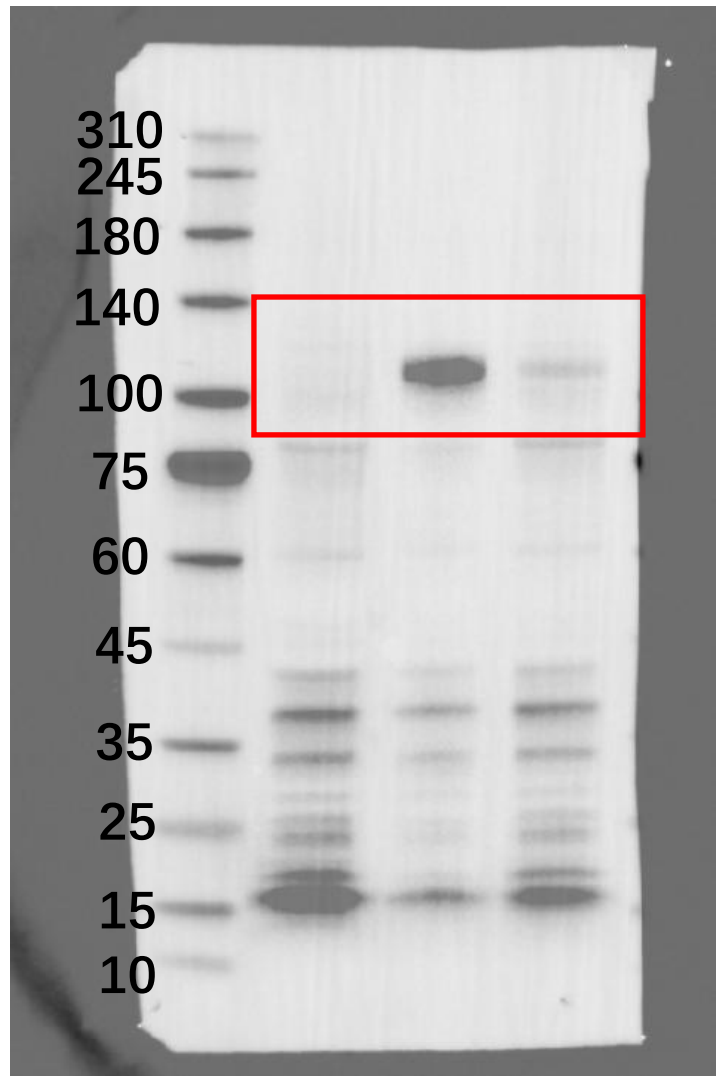

GAPDH： 37kDa

Repeat 1

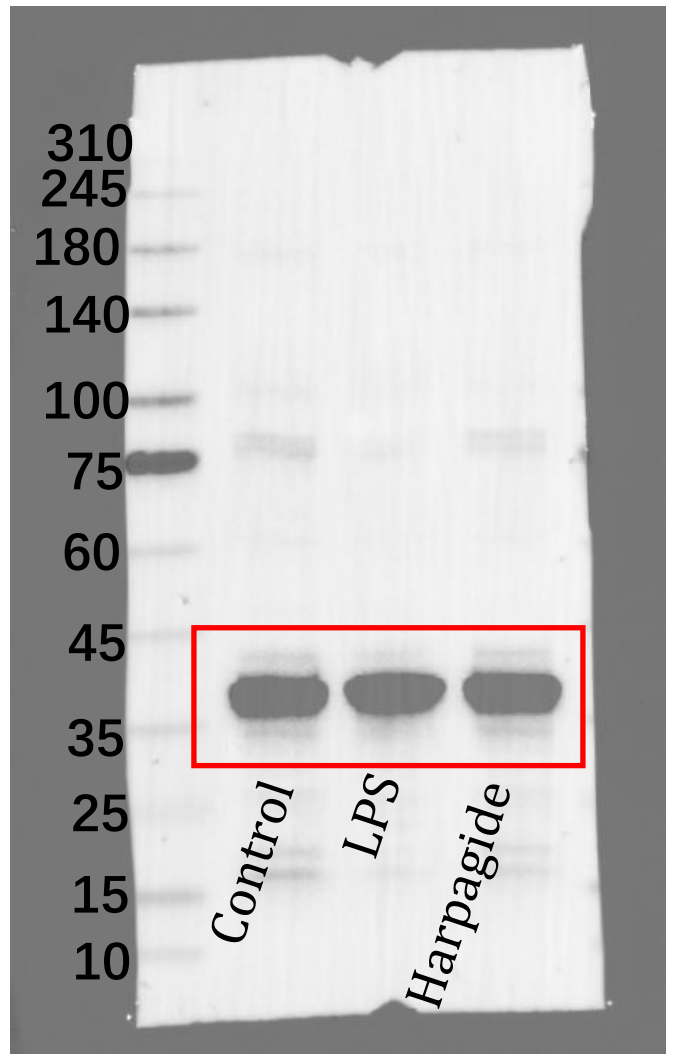

Repeat 2

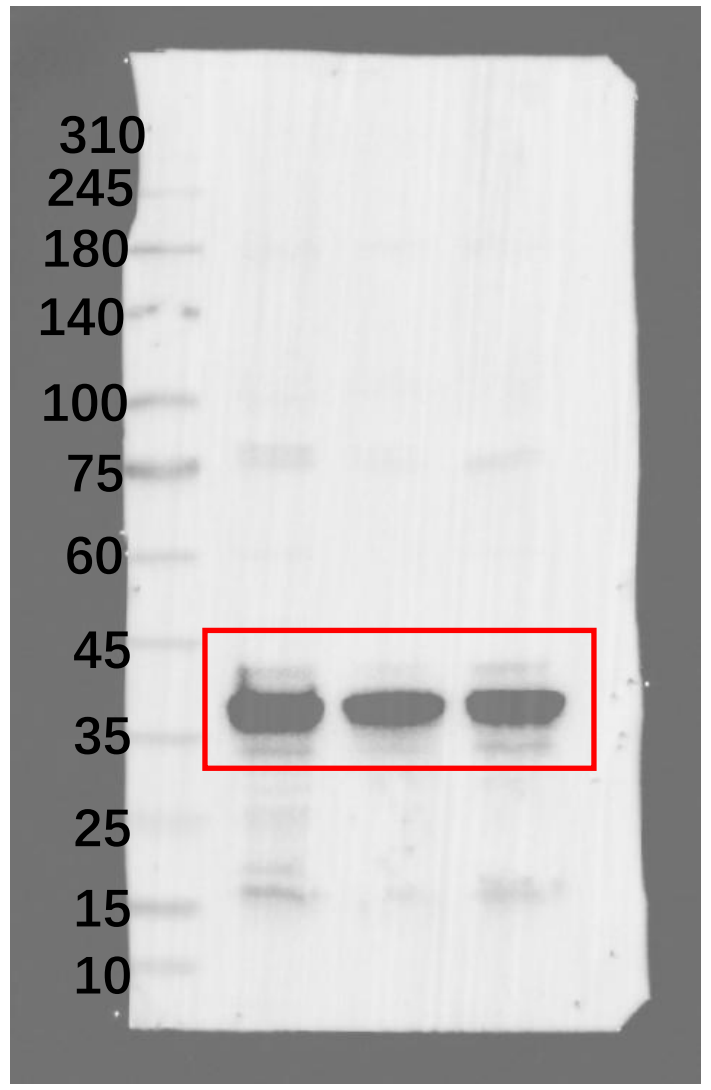

Repeat 3

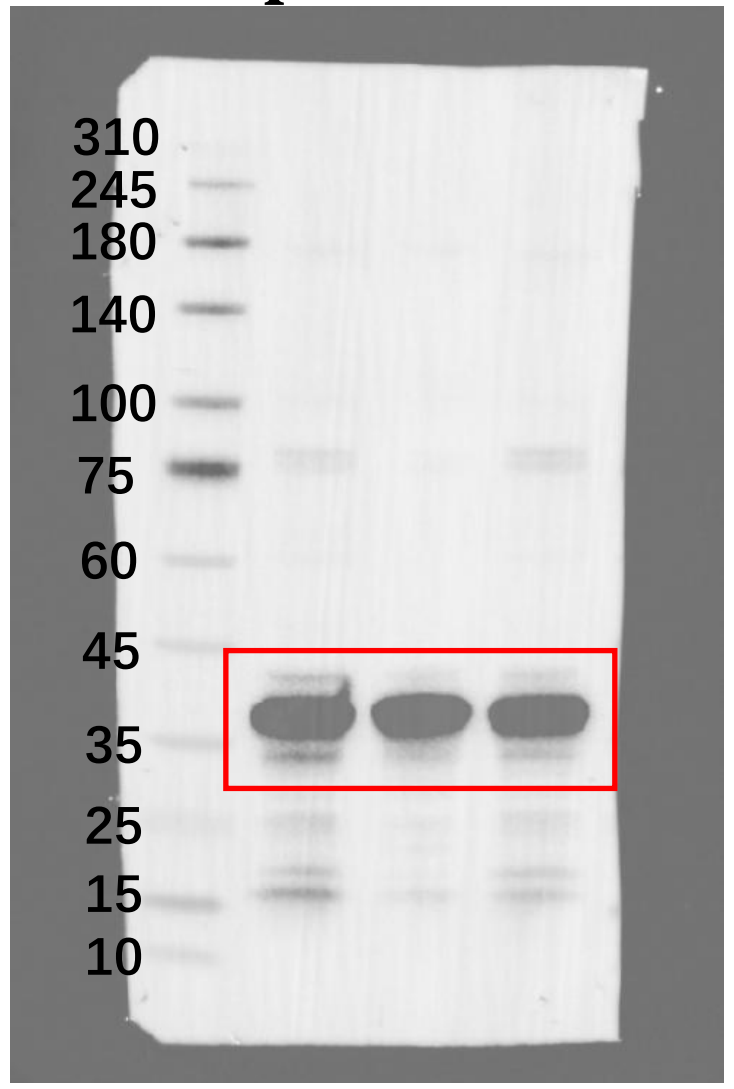

Full unedited gel/blot for Figure. 7B

NF-κB p65： 65kDa

GAPDH： 37kDa

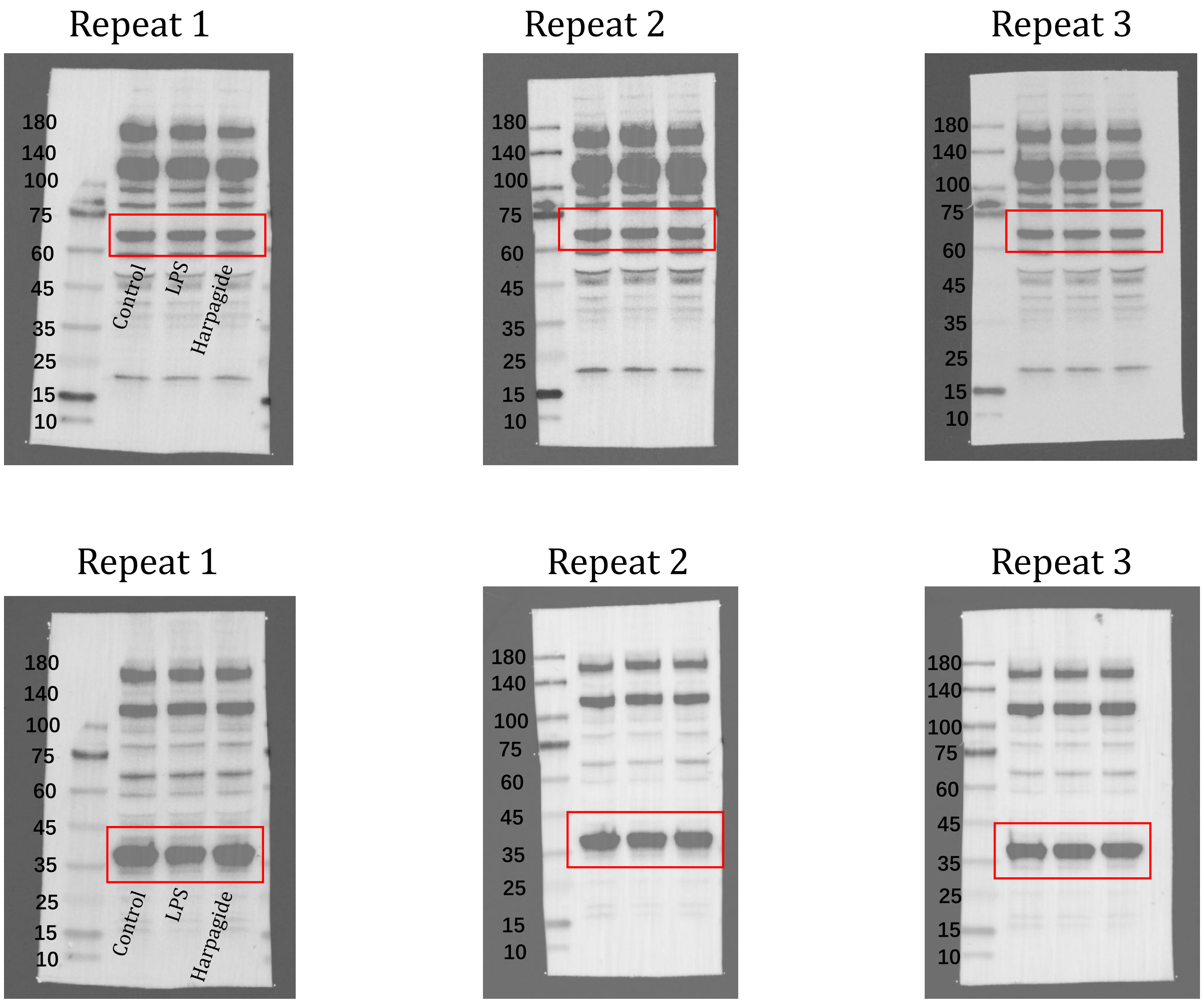

Supplement: Supplementary file 1 [file pone.0362296.s001.pdf]
